# Taxonomic review of the Pterostichini and Loxandrini fauna of New Caledonia (Coleoptera, Carabidae)

**DOI:** 10.3897/zookeys.147.1943

**Published:** 2011-11-16

**Authors:** Kipling W. Will

**Affiliations:** 1 ESPM Department and Essig Museum of Entomology, University of California, Berkeley, CA 94720

**Keywords:** Ground beetles, Pacific Island fauna

## Abstract

The generic-level taxa included in Pterostichini and Loxandrini from New Caledonia are reviewed and a key to genera and species provided. Two new genera are described, *Paniestichus* and *Abacophrastus*, with the following new species: *Paniestichus subsolianus*, *Abacophrastus millei*, *Abacophrastus chapes*, *Abacophrastus carnifex*, *Abacophrastus hobbit*, *Abacophrastus megalops*, *Abacophrastus reflexus* and *Abacophrastus bellorum*. *Abacoleptus curtus* new species, is described. *Notonomus irideus* and *Notonomus savesi* are moved to *Prosopogmus*. Four new species of *Prosopogmus* are described: *Prosopogmus koghisensis*, *Prosopogmus lescheni*, *Prosopogmus fortis* and *Prosopogmus aoupiniensis*. *Homalosoma griseolum* is moved to *Sphodrosomus*. *Cerabilia* is newly recorded from New Caledonia and the genus is moved from Platynini to Loxandrini and therefore is the first report of Loxandrini from New Caledonia. An apparent adventive from Australia, *Darodilia*, is newly reported from New Caledonia.

## Introduction

New Caledonia, including Grande Terre, Ile des Pins and the Loyalty Islands is one of the most biologically fascinating places on Earth. It is known for its highly diverse and endemic flora (Dawson 1981, Jaffré 1992) and fauna (Bauer and Vindum 1990, Chazeau 1993, Malm and Johanson 2008) and is listed as the world’s smallest biodiversity hotspot (Myers et al. 2000). Soon after the arrival of the French to New Caledonia, specimens began to make their way to Europe where they were eagerly described by taxonomists of the period (Chazeau 1993). Recently, a resurgent interest in New Caledonian biodiversity has been partly stimulated by the highly controversial notion that Grande Terre was completely submerged until the Oligocene (Murienne et al. 2005, Grandcolas et al. 2008). The corollary of this hypothesis is that the terrestrial and freshwater life on New Caledonia is entirely derived from recent dispersing individuals, or the alternative offered by Heads (2008), that small populations of organisms hopped between local ephemeral small islands until Grande Terre became emergent. Both scenarios conflict with evidence of a more ancient vicariance origin coincident with the breakup of Gondwana, estimated to have occurred around 80mya, and the apparent limited dispersal abilities of some New Caledonian taxa. The latter would seem to require persistent emergence of habitable land e.g., (Sharma and Giribet 2009). How the communities of plants and animals came to be assembled on New Caledonia as they are today remains unanswered. Darlington (1957: 535-537), long before the current debate about the oceanic or continental nature of the New Caledonia fauna, held that the pattern of vertebrates suggested dispersal was the sole mechanisms, but that “*New Caledonia too is supposed to be an old island, but its vertebrate fauna is not obviously old. It includes no ancient relicts…. The occurrence and geographical relationships of many invertebrates are not yet well known, and it is dangerous to generalize about them from the literature, but I know that carabid beetles (which I study) are highly differentiated and apparently old on Madagascar, New Zealand, and New Caledonia… The carabids therefore agree with the vertebrates up to a point but suggest that the New Caledonian fauna is old…”*.

A fundamental step toward revealing the biogeographic story is the discovery and description of species across many groups and reconstructing their phylogenetic relationships. New Caledonian carabid beetles are a significant part of the islands’ ecological community, but they remain rather poorly described and so have not yet been analyzed in biogeographical context.

Currently there are about 120 species of Carabidae recorded from New Caledonia, with the largest tribe being Cicindelini (24 species). However, the predominance of tiger beetles is due to a biased description effort rather than an actual numerical dominance of species in the fauna. Primarily based on material recently collected by researchers affiliated with institutions listed below and my own collecting, it is now apparent that the Pterostichini and Loxandrini (Pterostichini s.l. auctorum) comprise the most species rich carabid taxon on the islands with over 50 species. The single loxandrine genus *Cerabilia* Laport de Castlenau includes about half of those species.

No general taxonomic treatment of the pterostichine carabid beetles of New Caledonia has been published since Fauvel’s (1882a, b, c, 1903) overview work on the carabids of the island. Fauvel and various authors publishing on the carabid fauna of New Caledonia around the turn of the last century were working independently and had access to very limited material and so were not able to effectively place species in genera. In this contribution generic placement of species is made consistent with the current the classification and results of character analyses to be published elsewhere. New species and genera are described based on recently collected material and all available material and type specimens were bought together for this review. Although it is a significant advance from the time of Fauvel, the material available and number of recent collecting events is still too small to consider the New Caledonian pterostichine fauna to be well known. In particular, genera like *Prosopogmus* Chaudoir and *Cerabilia* that have many species with very limited ranges are likely to have many more species yet to be discovered. This contribution is strictly taxonomic in nature and genera are listed here as they emerge from the artificial key provided. A cladistic analysis and phylogeny of Pterostichitae is to be published elsewhere, but the generic concepts presented here are consistent with the preliminary analyses of that ongoing study.

## Methods

Dissection methods for male and female genitalia and defensive glands, measurements and descriptive terms follow (Liebherr and Will 1998, Will 2002). Images were taken using a Microptics XLT digital imagining system and subsequently edited to enhance clarity using standard image editing software.

Loans of material were kindly provided for this study by the following institutions and individuals: Queensland Museum, Brisbane, Geoff Monteith (QM); California Academy of Sciences, San Francisco, David Kavanaugh (CAS); Muséum National d’Histoire Naturelle, Paris, Theirry Deuve (MNHN); New Zealand Arthropod Collection, Auckland, Richard Leschen (NZAC); Essig Museum of Entomology, Berkeley (EMEC); Zoologische Staatssammlung, München, Martin Baehr and Michael Balke (ZMS), Staatliche Naturhistorische Sammlungen, Museum fuer Tierkunde, Olaf Jaeger, (MTD); Institut Royal des Sciences Naturelles de Belgique, Alain Drumont, (IRSNB); P.M. Giachino collection, Turin (GC).

Examined types are indicated by an exclamation point. Ocular ratio refers to the width over eyes/width between eyes. Standard body length (SBL) is the sum of the measurement of the head from the base of the labrum to the cervical sulcus, mid-length of the pronotum and length of left elytron from base to apex.

Database code numbers affixed as additional labels in the form of “EMEC######” are reported here and can be accessed, including mapping of georeferenced specimens, via the EMEC web pages at essigdb.berkeley.edu.

## Treatment of genera and species

### Genus Platycaelus

Blanchard, 1853:25

http://species-id.net/wiki/Platycaelus

Figs 1 [Fig F2] 3 43A

#### Type species.

*Platycaelus depressus* Blanchard 1853, by monotypy.

#### Description.

**Figure 2. F2:**
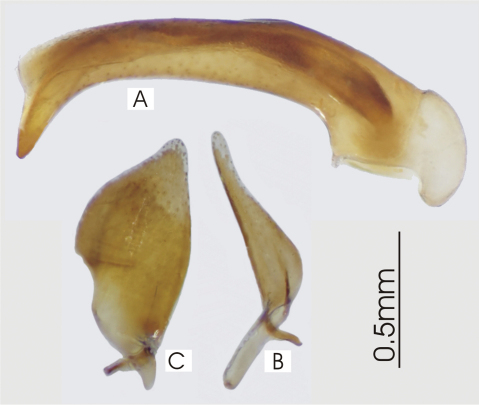
Male aedeagus of *Platycaelus melliei*, specimen from WA, Aus **A** right lateral view **B** right paramere **C** left paramere.

**Figure 3.**
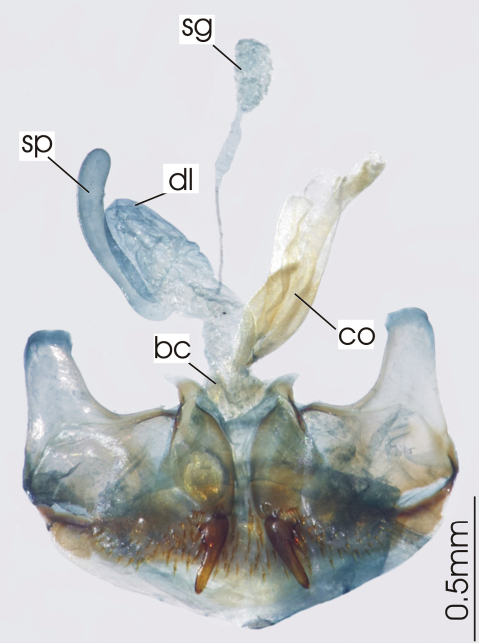
Female reproductive tract, ventral view. *Platycaelus melliei*. bc. bursa copulatrix, co. common oviduct, dl. dorsal lobe of bursa, sg. spermatheca gland sp. spermatheca.

*Head*. Clypeo-ocular sulci long, straight, very broadly and very shallowly impressed; mentum emarginate, sides divergent, paramedial pits small, deeply impressed; median tooth bifid; paraglossae small, without elongate setae at apex; ligular sclerite with two seta on apical margin; maxillary palpifer with one basal seta; antennae filiform, with three basal segments glabrous. *Thorax*. Pronotum quadrate, width across base slightly broader than apex, two marginal setae; pro-, meso- and metasterna glabrous; proepisternum with very shallow scattered punctulae; elytra free, border at base, nine well impressed striae, apicolateral plica large and visible, parascutellar stria long, impressed, not connected to stria 1, angular base of stria 1 well impressed, parascutellar punctues at base of stria 2, no discal punctures, intervals flat or scarcely convex; hind wing full; anterior tarsi of male with three basal segments expanded, ventrally squamous. *Abdomen*. Ventrites 3-6 without sulci; aedeagus (fig. 2) ostium dorsal, median lobe oriented left side up in repose; parameres attenuate with long narrow apex, both nearly of equal length; female reproductive tract (fig. 3) with dorsolateral bursal lobe, elongate spermatheca broadly attached laterally at base of bursal lobe, with appended gland attached near base of spermatheca, spermatheca without digitiform diverticulum near base, without spermathecal gland duct diverticulum.

**Figure 1.** Habitus image, *Platycaelus melliei*, specimen from WA, Aus.

#### New Caledonian species.

*Platycaelus melliei* (Montrouzier, 1860)

=*Feronia melliei* Montrouzier 1860

=*Poecilus chlaenioides* Macleay 1888

=*Feronia herbaceus* Chaudoir 1865

=*Feronia resplendens* Laporte de Castlenau 1867

*Platycaelus prolixus* (Erichson 1842)

=*Pterostichus prolixus* Erichson 1842

=*Chlaenioidius caledonicus* Tschitscherine 1901

=*Feronia funebris* Laporet de Castlenau 1867

=*Chlaenioidius planipennis* Macleay 1871

=*Poecilus sulcatulus* Macleay 1888

#### Exemplars of species examined.

*Platycaelus melliei*, *Platycaelus prolixus*, *Platycaelus poeciloides* (Chaudoir).

#### Generic Distribution:

 Australia, Moluka Islands, New Britain Island, New Caledonia, New Guinea and Tasmania.

#### Notes.

 These beetles are abundant and commonly collected in Australia. Based on my UV/MV light collections in Australia they are strong fliers. Most likely the presence of the two Australian species in New Caledonia is due to human transport or possibly dispersal.

### Genus Darodilia

Castelnau, 1868:157

http://species-id.net/wiki/Darodilia

**Figure 4.**
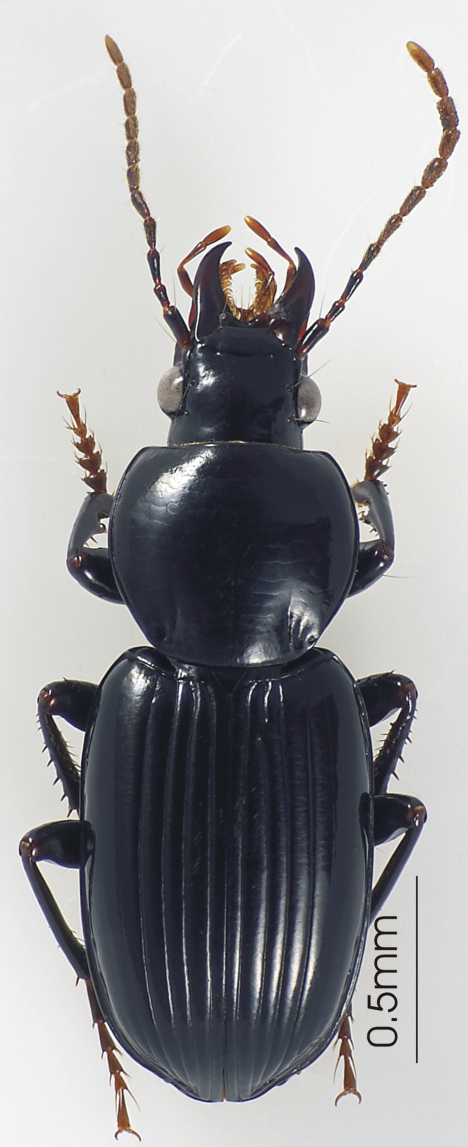
Habitus image, *Darodilia* undetermined species from New Caledonia.

Figs 4 [Fig F5] 6 44

#### Type species.

*Darodillia mandibularis* Castelnau 1868, by monotypy.

#### Description.

**Figure 5. F5:**
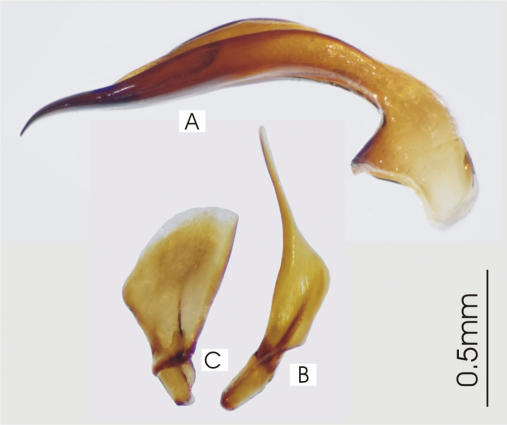
Male aedeagus of undetermined *Darodilia* species from New Caledonia **A** right lateral view **B** right paramere **C** left paramere.

**Figure 6.**
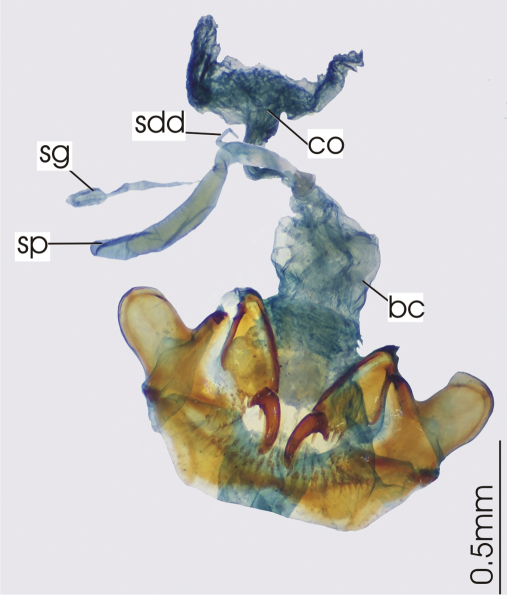
Female reproductive tract, ventral view. *Darodilia mandibularis*. bc. bursa copulatrix, co. common oviduct, sdd spermathecal digitiform diverticula, sg. spermatheca gland, sp. spermatheca.

*Head*. Clypeo-ocular sulci absent or present (present in some Australian species); mentum very shallowly emarginate, sides divergent, paramedial pits large, deeply impressed; median tooth simple, rounded; paraglossae small, without elongate setae at apex; ligular sclerite with two seta on apical margin; maxillary palpifer with one basal seta; antennae filiform, with three basal segments glabrous. *Thorax*. Pronotum orbiculate or cordate, two marginal setae; pro-, meso- and metasterna glabrous; proepisternum smooth or with deep longitudinal strigae (strigae in some Australian species); elytra free, border at base, striae 1-4, 8-9 impressed, 5-7 absent or scarcely marked, number of impressed striae in Australian species variable, very short tenth stria at level of plica, apicolateral plica large and visible, parascutellar stria impressed and continuous with stria 1, angular base of stria 1 absent, parascutellar punctures at base of stria 2, no discal punctures, intervals flat or scarcely convex; hind wing full; anterior tarsi of male with three basal segments expanded, ventrally squamous, all tarsi dorsally glabrous. *Abdomen*. Ventrites 3-6 transversely sulcate; aedeagus (fig. 5) ostium dorsal, median lobe oriented left side up in repose; right paramere long, tip attenuate, left short rounded at apex; female reproductive tract (fig. 6) without dorsolateral bursal lobe, elongate spermatheca broadly attached laterally at base of common oviduct, spermatheca with appended gland, spermatheca with digitiform diverticulum near base, without spermathecal gland duct diverticulum.

#### New Caledonia species.

*Darodilia* undetermined sp.

#### Exemplars of species examined.

*Darodilia longula* Tschitshérine, *Darodilia robusta* Sloane, *Darodilia mandibularis* castelnau, seven undetermined (probably undescribed) species of *Darodilia* from Australia and one species from New Caledonia.

#### Generic distribution.

 Australia and New Caledonian.

#### Notes.

 This genus has not been previously recorded from New Caledonia. I have seen two male specimens from La Foa, Pocquereux (approximate location 21°43'S, 165°54'E), 50m elevation and Koumac (approximate location 20°33'S, 164°16'E), 60m elevation, collected in February 2008 by P.M. Giachino (GC), and one male from Tiea Reserve, 21°07'S, 164°57'E, 30m, November 2001, collected by C. Burwell & G. Monteith (EMEC). These beetles are commonly collected in northern Australia and based on my UV/MV light collections in Australia they are strong fliers. Most likely the presence of this species in New Caledonia is due to human transport or dispersal. The specimens from New Caledonia appear to be conspecific with others from Australia I have studied. However, as the genus needs revised it is not possible to confidently identify the specimens without recourse to the types.

### Genus Pseudoceneus

Tschitschérine, 1890:164

http://species-id.net/wiki/Pseudoceneus

Figs 7 [Fig F8] 9

#### Type species.

*Argutor holomelanus* Tschitschérine 1890, see Moore et al. 1987 for details on type.

#### Description.

*Head*. Clypeo-ocular sulci absent; mentum emarginate, sides divergent, paramedial pits moderately large, deeply impressed; median tooth bifid; paraglossae small, without elongate setae at apex; ligular sclerite with two seta on apical margin; maxillary palpifer with one basal seta; antennae filiform, with three basal segments glabrous. *Thorax*. Pronotum quadrate, slightly transverse to slightly elongate, two marginal setae; pro-, meso- and metasterna glabrous; proepisternum smooth; elytra free, border at base, nine fully impressed striae, very short tenth stria at level of plica, apicolateral plica small and visible, parascutellar stria long, well impressed, not continuous with stria 1, angular base of stria 1 present, parascutellar punctures absent, three discal punctures in interval 3, intervals flat or convex; hind wing full; anterior tarsi of male with three basal segments expanded, ventrally squamous, all tarsi dorsally glabrous. *Abdomen*. Ventrites 3-6 without transverse sulci; aedeagus (fig. 8) ostium dorsal, oriented left side up in repose; right paramere small, narrow attenuate, left short, rounded point at apex; female reproductive tract (fig. 9) without dorsolateral bursal lobe, elongate spermatheca broadly attached laterally at base of common oviduct, spermatheca with appended gland, without spermatheca, without duct digital diverticulum, without spermathecal gland duct diverticulum.

**Figure 7.**
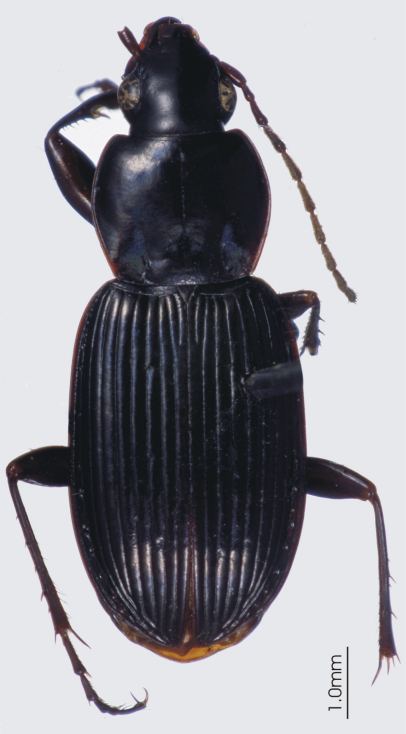
Habitus image, *Pseudoceneus numeensis* holotype.

**Figure 8. F8:**
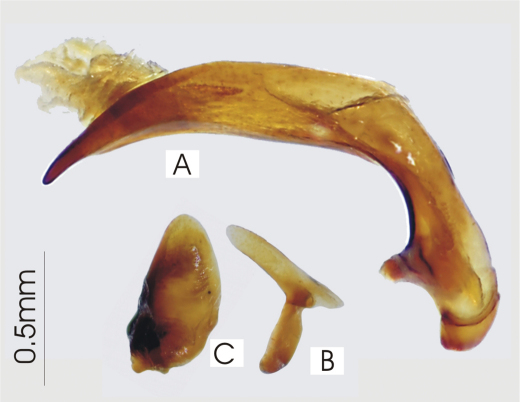
Male aedeagus of *Pseudoceneus numeensis*, holotype **A** right lateral view **B** right paramere **C** left paramere.

**Figure 9.**
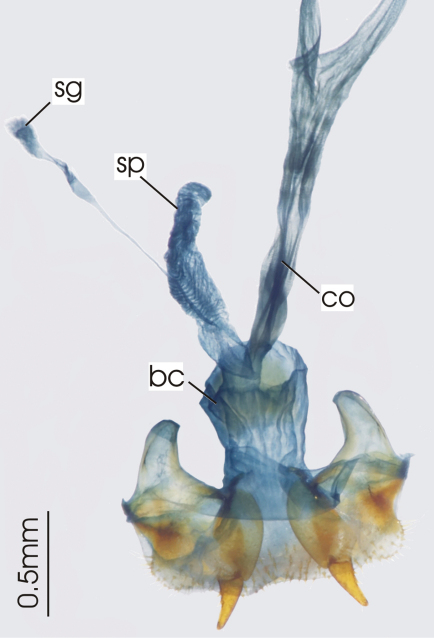
Female reproductive tract, ventral view. *Pseudoceneus sollicitus*. bc. bursa copulatrix, co. common oviduct, sg. spermatheca gland, sp. spermatheca.

#### New Caladonian species.

*Pseudoceneus numeensis* Fauvel 1903 [type IRSNB!]

#### Exemplars of species examined.

*Pseudoceneus beatricis* Giachino, *Darodilia iridescens* (Castelnau), *Darodilia sollicitus* (Erichson), *Pseudoceneus numeensis* Fauvel and numerous specimens including several apparently undescribed species from Australia.

#### Generic distribution.

 Australia, New Caledonia, Norfolk Island and Tasmania.

#### Notes.

 This genus was treated recently by Giachino (2005), however, he did not include *Pseudoceneus numeensis* in his study. Based on that paper and specimens available to me, I cannot confidently identify many individuals from Australia and some of those are very similar to *Pseudoceneus numeensis*. Based on my preliminary study there is a significant amount of continuous variation in gross features, such as pronotal form and elytral structures, while there is little and very subtle, variation in male genitalia. This genus requires revision based on a large sample of material and probably will require DNA sequence data for full resolution of the species boundaries. Given the material I have seen, *Pseudoceneus numeensis* appears distinct from typical individuals of other currently name species, but is probably conspecific with a widespread and commonly collected species in Queensland, Australia and thus likely represents a recent introduction or dispersal from Australia to New Caledonia.

### Genus Prosopogmus

Chaudoir, 1865: 92

http://species-id.net/wiki/Prosopogmus

**Figure 10.**
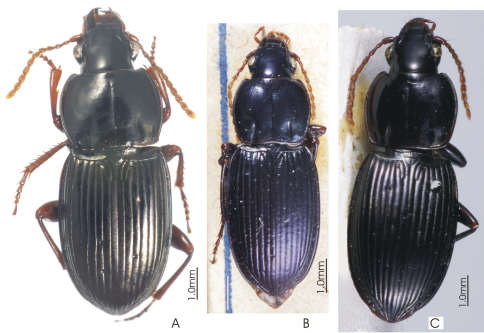
Habitus images of **A** *Prosopogmus lescheni* **B** *Pseudoceneus irideus*, holotype **C** *Pseudoceneus savesi*, holotype.

Figs 10 [Fig F11] [Fig F12] 13 45

#### Type species.

*Feronia impressifrons* Chaudoir 1865, by monotypy.

#### Description.

**Figure 11. F11:**
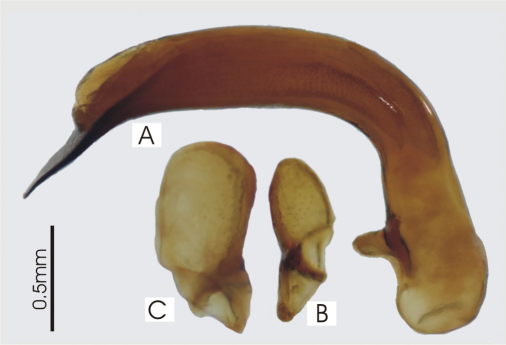
Male aedeagus of *Prosopogmus koghisensis*, holotype **A** right lateral view **B** right paramere **C** left paramere.

**Figure 12. F12:**
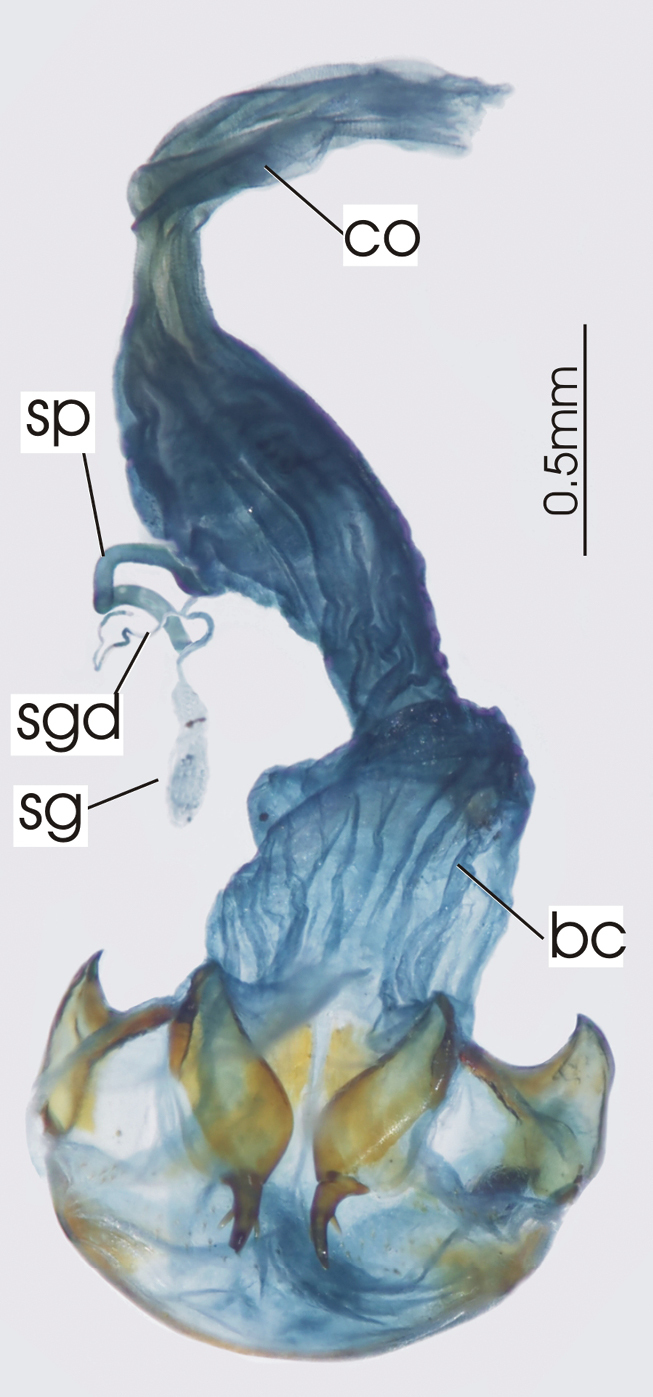
Female reproductive tract, ventral view. *Prosopogmus koghisensis*. bc. bursa copulatrix, co. common oviduct, sg. spermatheca gland, sgd. spermathecal gland diverticula, sp. spermatheca.

**Figure 13.**
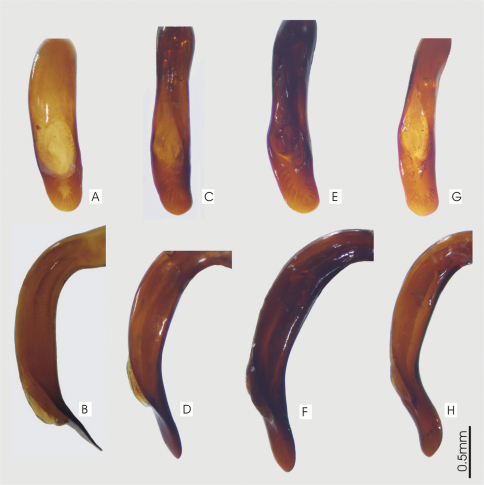
Blade of aedeagus, dorsal view **(A, C, E, G)** right lateral view **(B, D, F, H)** for *Prosopogmus koghisensis*. **A, B** *Pseudoceneus lescheni*. **C–F** *Pseudoceneus fortis*. **G, H**.

*Head*. Clypeo-ocular sulci deeply impressed, divergent; mentum moderately or shallowly emarginate, sides divergent, paramedial pits distinct and deep, or absent; median tooth emarginate or bifid; paraglossae small, without elongate setae at apex; ligular sclerite with two seta on apical margin; maxillary palpifer with one basal seta; antennae filiform, with three basal segments glabrous. *Thorax*. Pronotum subcordiform, trapezoidal or quadrate, two marginal setae; pro-, meso- and metasterna glabrous; proepisternum smooth; elytra free or fused, bordered at base, nine fully impressed striae, apicolateral plica large and visible, parascutellar stria present, angular base of stria 1 present or absent, parascutellar setigerous punctures present at base of stria 2, three discal punctures confined to third intervals, posterior pair touching or situated near stria 2, intervals flat or convex; hind wing full or reduced (reduced in New Caledonian species); anterior tarsi of male with three basal segments expanded and squamose beneath, all tarsi dorsally glabrous. *Abdomen*. Ventrites 3-6 transversely sulcate; aedeagus (Figs 11, 13) ostium dorsal, median lobe oriented left side up in repose; parameres conchoid; female reproductive tract (fig. 12) without dorsolateral bursal lobe, bursa gooseneck form, elongate spermatheca broadly attached on posterior face of bursa, spermatheca with appended gland, without duct digital diverticulum, with elongate spermathecal gland duct diverticulum.

#### New Caladonian species.

*Prosopogmus savesi* (Fauvel 1882) new combination [type IRSNB!]

*=Notonomus savesi* Fauvel 1882

*Prosopogmus irideus* (Fauvel 1903) new combination [type IRSNB!]

*=Notonomus irideus* Fauvel 1903

*Prosopogmus koghisensis* Will, sp. n.

*Prosopogmus lescheni* Will, sp. n.

*Prosopogmus fortis* Will, sp. n.

*Prosopogmus aoupiniensis* Will, sp. n.

#### Exemplars of species examined.

 All six species from New Caledonia and numerous species from Australia.

#### Generic distribution.

 Australia, Fiji(?), Lord Howe Island, Maluku Islands, New

Caledonia, New Guinea, New Zealand and Tasmania.

#### Notes.

 This is a species-rich genus, with about 25 species described from Australia. However, this is probably less than one third of the true diversity. In all areas where it occurs, but particularly in Australia and New Guinea, this taxon needs revision. The four species described here are based on a modest number of specimens and collecting events; there is certain to be more new species in New Caledonia to be discovered. The two species described by Fauvel in *Notonomus*, *Pseudoceneus irideus* (fig. 10B) and *Pseudoceneus savesi* (fig. 10C), share no generic-level characters with that genus. They have the following features found in Euchroina and/or *Prosopogmus* (a combination of plesiomorphic and synapomorphic states), which are not found in *Notonomus*: Clypeo-ocular sulci deeply impressed; abdominal ventrites 3-6 transversely sulcate; and spermathecal gland with duct diverticulum.

### Prosopogmus koghisensis

Will sp. n.

urn:lsid:zoobank.org:act:AD615BD5-1073-4DF7-B84E-A6A2C7D5B25D

http://species-id.net/wiki/Prosopogmus_koghisensis

Figs 11 12 13A-B 45A

#### Type locality.

 New Caledonia, Southern Province, Mt. Koghis, 22°10'28"S, 166°30'48"E, 700m, rainforest.

#### Type material.

 Holotype: male (EMEC81200), “22°10'28"S, 166°30'48"E, NEW CALEDONIA Prov. Sud Mt.Koghis, 700m el. Coll.KWill 12.iii.2007, headlamp search", genitalia dissected, deposited MNHN. Paratypes: same data as holotype,1 female (EMEC81206) [NZAC], 1 female (EMEC81202) [QM], 1 female (EMEC81204) [MNHN], 1 male (EMEC81205) and 2 females (EMEC81201 EMEC81203) [EMEC].

#### Description.

*Size*. Overall length (sbl) 8.0-9.0mm, greatest width over elytra 3.1-3.8mm. *Color*. Dorsal and ventral surfaces black. Legs, mouthparts, and antennae slightly or noticeably paler, piceous to rufopiceous. *Luster*. Dorsally and ventrally shiny, elytra slightly duller. *Iridescence*. Elytra and ventral surface of body with slight spectral iridescence, otherwise lacking. *Head*. Dorsal microsculpture with microlines very shallow, visible at 50x magnification, sculpticells isodiametric or somewhat irregular mesh, clypeal-ocular sulci deeply impressed, linear, divergent, extended to anterior supraorbital seta, ocular ratio 1.35-1.42, eyes average size, rounded. Labrum with anteromedial depression. Antennae: Overall length short, reaching back 2/3 length of pronotum, antennomeres 5-11 nearly quadrate. *Thorax*. Pronotum nearly quadrate, sides evenly rounded from apex to near base, only a very slight straightening near base, marginal bead continuous from apex to base, not extended along basal margin, basal margin not bordered, anterior margin nearly straight with anterior angles scarcely produced, hind angles (fig. 45A) obtuse and rounded, inner basal impression linear and well impressed, outer impression absent, seta at hind angle about 3x pore width forward of basal margin 1x pore width medial of lateral channel. Dorsal surface shiny, microsculpture absent from disc, visible as irregular mesh of microlines laterally and basally at 50x magnification. Elytral striae complete, well impressed and impunctate. Parascutellar stria present. Angular base of stria 1 absent. Elytra moderately shiny, microsculpture visible at 20x as stretched mesh of microlines. Metacoxal sulcus straight and nearly reaching lateral end of coxa. *Abdomen*. Last abdominal ventrite with very broad, thick apical bead. Male aedeagus (fig. 13A-B) slightly curved to right in dorsal view, tip broadly, uniformly rounded.

#### Etymology.

 The name, treated as an adjective, is from the type locality, Mt.Koghis.

### Prosopogmus lescheni

Will sp. n.

urn:lsid:zoobank.org:act:90652B8F-7308-45A3-9DB5-D5956A2D72BC

http://species-id.net/wiki/Prosopogmus_lescheni

Figs 10 13C-F 45B

#### Type locality.

 New Caledonia, Southern Province, trail to Plateau de Dogny, 21°37'15"S, 165°52'29"E, 870m.

#### Type material.

 Holotype: male (EMEC81207), “21°37'15"S/165°52'29"E NEW CALEDONIA:Prov. Sud trail to Plateau de Dogny 870m. 15.iii.2007 K.Will", deposited MNHN. Paratypes: same data as holotype 2 males (EMEC81208, EMEC81209) and 1 female (EMEC81211) [EMEC]. “21°33'49"S/165°45'37"E NEW CALEDONIA Prov.Sud. Col d'Amieu 390m el. 16:iii:2007 Coll. R.Leschen" 3 males (EMEC81212, EMEC81213, EMEC81214) and 1 female (EMEC60970) [NZAC]. “21°34'29"S/165°45'19"E NEW CALEDONIA:Prov. Sud Col d'Amieu, 510m el. 510m 16.iii.2007 K.Will", 1 male (EMEC81210) [EMEC] and 1 female (EMEC81215) [QM]. “NEW CALEDONIA 21°34'S/165°46'E Table Unio road, 600m 14 Nov2000. Bouchard, Burewell, Monteith, 9934", 1 male (EMEC81216) [QM]. “NEW CALEDONIA, Pic Ningua summit, 1350m, 21°47.8'S 166°8.3'E, collected by pitfall trap, in primary montane rainforest, site L, January 1994, G.Hunt collector", 1 male (EMEC80944) and 1 female (EMEC80940) [CAS] , with same data except dates as follows: February 1993- 2 males (EMEC80942, EMEC80943) and 1 female (EMEC80941); March 1993- 5 males (EMEC80951, EMEC80952, EMEC80953, EMEC80954, EMEC80955); 4 April 1993- 1 female (EMEC80945); September 1993- 4 males (EMEC80947, EMEC80948, EMEC80949, EMEC80950) and 1 female (EMEC80946) [CAS].

#### Description.

*Size*. Overall length (sbl) 7.5–8.8mm, greatest width over elytra 2.8–3.5mm. *Color*. Dorsal and ventral surfaces black some individuals with a slight bronze reflex on elytra. Legs, mouthparts, and antennae slightly or noticeably paler than ventral surface of body, piceous to rufopiceous or concolorous with ventral surface and nearly black. *Luster*. Dorsally and ventrally moderately shiny to slightly dull, elytra usually slightly duller. *Iridescence*. Elytra and ventral surface of body with no or only a very slight spectral iridescence, otherwise lacking. *Head*. Dorsal microsculpture with microlines well impressed, readily visible at 30x magnification, sculpticells isodiametric or slightly irregular, forming mesh, clypeal-ocular sulci deeply impressed, linear, divergent, ended short of anterior supraorbital seta, ocular ratio 1.38-1.45, eyes average size, rounded prominent. Labrum flat. Antennae: Overall length moderately long, reaching base of pronotum, antennomeres 5-11 elongate. *Thorax*. Pronotum nearly quadrate, sides evenly rounded from apex to near base, marginal bead continuous from apex to base, not extended along basal margin, ended at hind angle which forms a distinct angular jag, anterior margin slightly emarginate with anterior angles moderately produced, inner basal impression linear and well impressed, outer impression shallowly or deeply impressed, area laterad of hind outer impression convex, hind margin between basal impressions with more or less distinctly impressed border, seta at hind angle not more than one pore width forward of basal margin and in contact with lateral channel. Dorsal surface moderately shiny, microsculpture readily visible as irregular mesh of microlines at 20x magnification. Elytral striae complete, well impressed and impunctate. Parascutellar stria and angular base of stria 1 present. Elytra moderately shiny, microsculpture readily visible at 20x as mesh of microlines. Metacoxal sulcus straight or slightly arcuate and ended well before lateral end of coxa. *Abdomen*. Last abdominal ventrite with narrow, light apical bead. Male aedeagus (fig. 13C-F) in dorsal view, nearly straight to distal edge of ostium and then curved right, degree of curvature varies between individuals, tip rounded.

Etymology. A noun in the genitive case for the collector of part of the type series and excellent coleopterist Richard Leschen (NZAC).

### Prosopogmus fortis

Will sp. n.

urn:lsid:zoobank.org:act:684EC79F-C9C1-48A8-949D-01337A3B8B76

http://species-id.net/wiki/Prosopogmus_fortis

Figs 13G–H

#### Type Locality.

 New Caledonia, Southern Province, Pic Ningua, 21°47.8'S, 166°8.3'E, 1350m., pitfall trap in primary montane rainforest, 19 February 1995, collector G.Hunt.

#### Type material.

 Holotype, male (EMEC80961), “NEW CALEDONIA, Pic Ningua summit, 1350m, 21°47.8'S, 166°8.3'E21°47.8'S 166°8.3'E, collected by pitfall trap, in primary montane rainforest, site L, 19 February 1995, G.Hunt collector” deposited MNHN. Paratypes, same data as holotype except dates, as follows: 9 January 1993- 2 males (EMEC80959, EMEC80960); 24 January 1994-1 male (EMEC80958) and 1 female (EMEC80957) [CAS].

#### Description.

*Size*. Overall length (sbl) 7.5-8.8mm, greatest width over elytra 2.8-3.5mm. *Color*. Dorsal and ventral surfaces black some individuals with a slight bronze reflex from elytra. Legs, mouthparts, and antennae slightly or noticeably paler than ventral surface of body, piceous to rufopiceous or concolorous with ventral surface and then nearly black. *Luster*. Dorsally and ventrally moderately shiny to slightly dull, elytra usually slightly duller. *Iridescence*. Elytra and ventral surface of body with no or only a very slight spectral iridescence, otherwise lacking. *Head*. Dorsal microsculpture with microlines well impressed, readily visible at 30x magnification, sculpticells isodiametric or slightly irregular, forming mesh, clypeal-ocular sulci deeply impressed, linear, divergent, ended short of anterior supraorbital seta, ocular ratio 1.38-1.45, eyes average size, rounded prominent. Labrum flat. Antennae: Overall length moderately long, reaching base of pronotum, antennomeres 5-11 elongate. *Thorax*. Pronotum nearly quadrate, sides evenly rounded from apex to near base, marginal bead continuous from apex to base, not extended along basal margin, ended at hind angle which forms a distinct angular jag, anterior margin slightly emarginate with anterior angles moderately produced, inner basal impression linear and well impressed, outer impression shallowly or deeply impressed, area laterad of hind outer impression convex, hind margin between basal impressions with more or less distinctly impressed border, seta at hind angle not more than one pore width forward of basal margin in contact with lateral channel. Dorsal surface moderately shiny, microsculpture readily visible as irregular mesh of microlines at 20x magnification. Elytral striae complete, well impressed and impunctate. Parascutellar stria and angular base of stria 1 present. Elytra moderately shiny, microsculpture readily visible at 20x as mesh of microlines. Metacoxal sulcus straight or slightly arcuate and ended well before lateral end of coxa. *Abdomen*. Last abdominal ventrite with narrow, light apical bead. Male aedeagus (fig. 13G-H) in dorsal view nearly straight to distal edge of ostium and then curved right, tip rounded.

#### Etymology.

 Latin *fortis*, treated as an adjective, alludes to the well impressed basal impressions of the pronotum.

### Prosopogmus aoupiniensis

Will sp. n.

urn:lsid:zoobank.org:act:49ADDB7D-BEA2-4CB1-A6D6-A1FFE0B5BF72

http://species-id.net/wiki/Prosopogmus_aoupiniensis

#### Type locality.

 New Caledonia, Northern Province, 21°11'S, 165°16E, 1000m.

#### Type material.

 Holotype, female (EMEC80956). “NEW CALEDONIA11665 21°11'S, 165°16'E Aoupinie, summit, 1000m, 20Oct2004. G.Monteith, pyrethrum, trees & logs” deposited MNHN.

#### Description.

*Size*. Overall length (sbl) 8.3mm, greatest width over elytra 3.3mm. *Color*. Dorsal and ventral surfaces black, legs, mouthparts, and antennae noticeably paler rufopiceous. *Luster*. Dorsally and ventrally moderately shiny. *Iridescence*. Elytra and ventral surface of body with slight spectral iridescence, otherwise lacking. *Head*. Dorsal microsculpture with microlines shallowly impressed, scarcely visible at 50x magnification, sculpticells isodiametric or slightly irregular, forming mesh, clypeal-ocular sulci deeply impressed, linear, divergent, ended just short of anterior supraorbital seta, ocular ratio 1.35, eyes small, rounded very prominent. Labrum with anteromedial depression. Antennae: Overall length short, reaching back 2/3 length of pronotum, antennomeres 5-11 only slightly elongate. *Thorax*. Pronotum elongate and relatively narrow, sides evenly, shallowly rounded from apex to just before base, then nearly straight to hind angles, marginal bead continuous from apex to base, not extended along basal margin, ended at hind angle which forms a low, rounded jag, anterior margin straight, anterior angles not produced and tight to head, inner basal impression linear and well impressed, outer impression absent, hind margin laterad basal impressions with more or less distinctly impressed border, seta at hind angle not more than one pore width forward of basal margin and just touching lateral channel. Dorsal surface shiny, microsculpture absent from disc, visible as irregular mesh of microlines laterally and basally at 50x magnification. Elytral striae complete, well impressed and impunctate. Parascutellar stria present. Angular base of stria 1 absent. Elytra moderately shiny, microsculpture visible at 20x as stretched mesh of microlines. Metacoxal sulcus straight and nearly reaching lateral end of coxa. *Abdomen*. Last abdominal ventrite with very broad, thick apical bead. Male unknown. Female reproductive tract not studied.

#### Etymology.

 Name, treated as an adjective, is from the type locality, Mt. Aoupinie.

### Genus Paniestichus

Will gen n.

urn:lsid:zoobank.org:act:0808BE48-6A27-466F-B74A-F5E5AEA60EFC

http://species-id.net/wiki/Paniestichus

**Figure 14.**
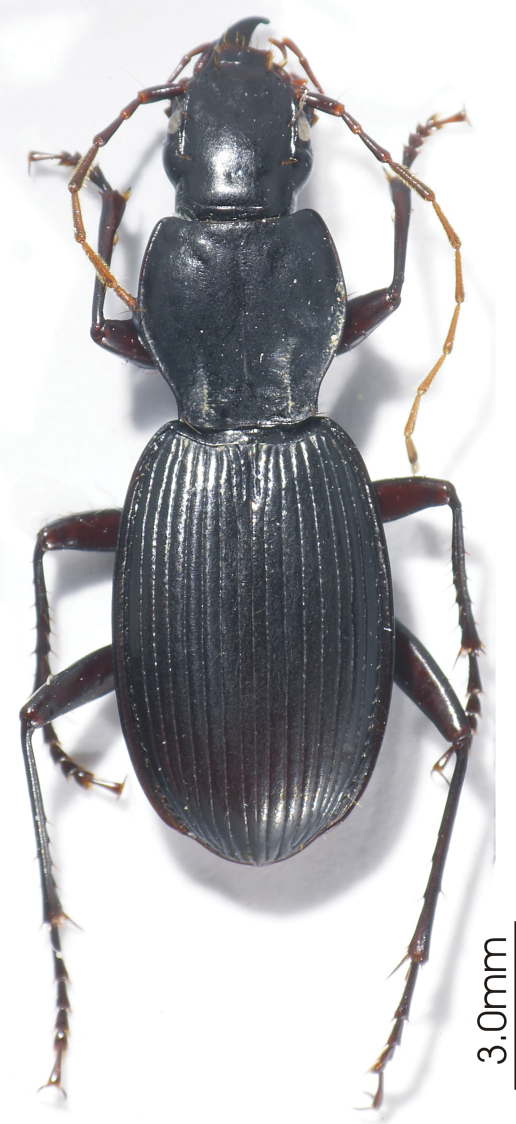
Habitus image, *Paniestichus subsolianus*, Holoytype.

Figs 14 [Fig F15] 16

#### Type species.

*Paniestichus subsolianus* Will sp. n.

#### Generic Description.

**Figure 15. F15:**
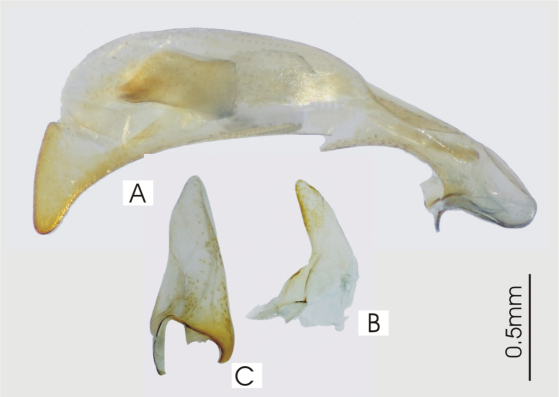
Male aedeagus of *Paniestichus subsolianus*. Teneral specimen **A** right lateral view **B** right paramere **C** left paramere.

**Figure 16.**
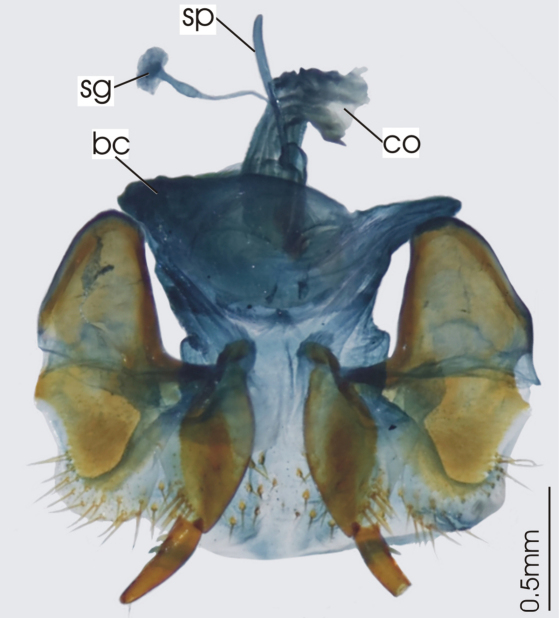
Female reproductive tract, ventral view. *Paniestichus subsolianus*, holoytype. bc. bursa copulatrix, co. common oviduct, sg. spermatheca gland, sp. spermatheca.

*Head*. Clypeo-ocular sulci absent, clypeo-ocular region with very shallow, broad depression; mentum deeply emarginate, sides nearly parallel, paramedial pits very large, diameter nearly full length from base of tooth to base of mentum, very deeply impressed; median tooth bifid; paraglossae long, without elongate setae at apex; ligular sclerite with two subapical setae; maxillary palpifer with two basal and one medial seta; antennae filiform, with three basal segments glabrous. *Thorax*. Pronotum cordate, two marginal setae; pro-, meso- and metasterna with short scattered pubescence; proepisternum smooth; elytra fused, border at base, nine fully impressed striae, apicolateral plica a small vestige, externally visible, parascutellar stria long, very shallowly impressed, not continuous with stria 1, angular base of stria 1 present, parascutellar punctures absent, no discal punctures, intervals flat; hind wing reduced; anterior tarsi of male with three basal segments not expanded, ventrally glabrous, all tarsi dorsally glabrous. *Abdomen*. Ventrites 3-6 without transvers sulci; aedeagus (fig. 15, teneral male and slightly damaged dissection) ostium dorsal, oriented left side up in repose; right paramere small, elongate and bluntly rounded, left elongate conchoid, rounded point at apex; female reproductive tract (fig. 16) without dorsolateral bursal lobe, elongate spermatheca broadly attached laterally at base of common oviduct with a small pouch on bursa near base of spermatheca, spermatheca with appended gland, without duct digital diverticulum, without spermathecal gland duct diverticulum.

#### Etymology.

*Paniestichus* is a compound word, based on the name of the type locality of the type species, Mt. Panei, and the common termination in Pterostichini –stichus. It is treated as a Latin masculine.

### Paniestichus subsolianus

Will sp. n.

urn:lsid:zoobank.org:act:52DC28EF-BB2B-49C6-95BA-BB22861223D9

http://species-id.net/wiki/Paniestichus_subsolianus

#### Type locality.

 New Caledonia, Northern Province, Mt. Panié, 1350 m.

#### Type material.

 Holotype, female (EMEC80968), “New Caledonia, North Prov. Mt. Panié, 1350 m. 8-9.11.2001 leg. Balke & Wewalka (NC16), 320DNA M. Balke”, returned to ZSM, to be hand carried and integrated into the MNHN collection for deposition by M. Balke. Paratype, male (teneral), same locality data as holotype except 1400m (NC 19), [ZSM] (EMEC80969).

#### Description.

*Size*. Overall length (sbl) 14.2-15.2mm, greatest width over elytra 5.2-5.4mm. *Color*. Dorsal and ventral surfaces black, legs, mouthparts, and antennomeres 1-4 black to piceous, antennomeres 5-11 paler, slightly infuscated. *Luster*. Dorsal and ventral surfaces moderately shiny. *Iridescence*. Elytra with distinct spectral iridescence. Ventral surface of body with slight spectral iridescence. *Head*. Dorsal microsculpture with microlines hardly visible at 50x magnification, sculpticells isodiametric or slightly irregular, forming mesh, clypeal-ocular sulci not impressed, represented as broad shallow depressions, ocular ratio 1.2-1.3, eyes very small, rounded, not prominent, post-ocular orbits relatively very large, including genal region more than 4x size of eye. Labrum anterior margin straight. Antennae: Overall length very long, antennomeres 9-11 reaching beyond base of pronotum, antennomeres very elongate. *Thorax*. Pronotum elongate cordate, sides shallowly rounded and convergent to near base and then very slightly sinuate and straight to base, marginal bead continuous from apex to base and across entire basal margin, hind angles about right angled, anterior margin very shallowly emarginate, anterior angles scarcely produced, inner basal impressions broad, shallow, not well impressed, slightly divergent, outer impression lacking, seta at hind angle in marginal bead. Dorsal surface moderately shiny, microsculpture not visible at 50x magnification. Elytral striae complete, shallowly impressed except laterally and apically, shallowly crenulate. Elytra moderately shiny, microsculpture not visible at 50x. Metacoxal sulcus straight and ended near lateral end of coxa. *Abdomen*. Last abdominal ventrite with very narrow, light apical bead.

#### Etymology.

 The specific epithet is a compound word from the Latin *sub* (below) and *solianus* (of the sun), alluding to the presumed hypogenous life history of this species.

### Genus Abacoleptus

Fauvel, 1903:232

http://species-id.net/wiki/Abacoleptus

**Figure 17.**
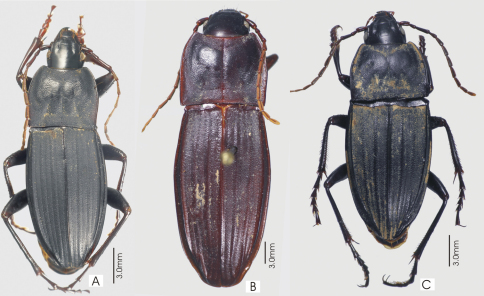
Habitus images **A** *Abacoleptus carinatus* **B** *Abacoleptus paradoxus*, holotype **C** *Abacoleptus curtus*, holotype.

Figs 17 [Fig F18] [Fig F19] 20

#### Type species.

*Abacoleptus carinatus* Fauvel 1903, by monotypy.

#### Description.

**Figure 18. F18:**
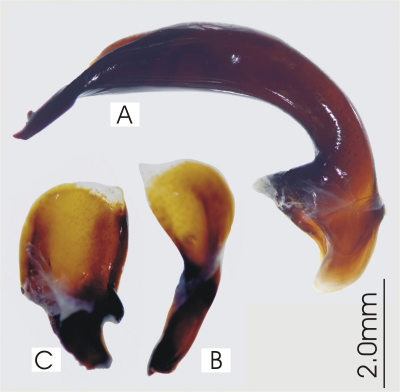
Male aedeagus of *Abacoleptus carinatus* **A** right lateral view **B** right paramere **C** left paramere.

**Figure 19. F19:**
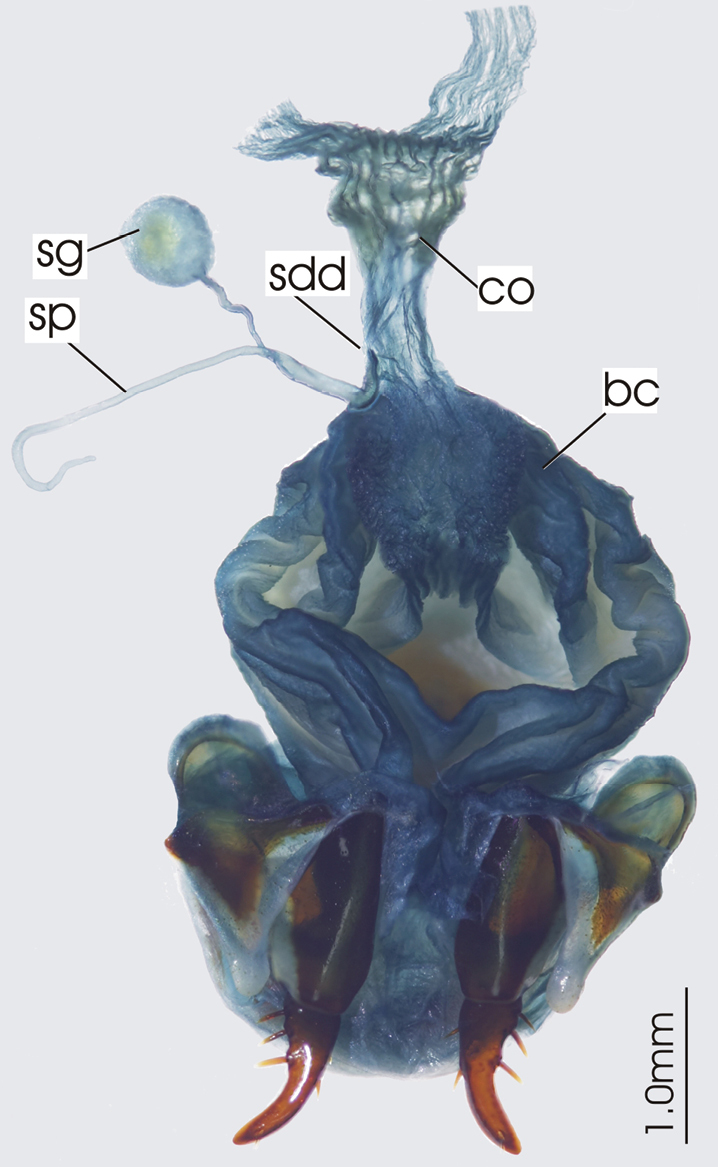
Female reproductive tract, ventral view. *Abacoleptus carinatus*. bc. bursa copulatrix, co. commonoviduct, spermathecal digitiform diverticula, sg. spermatheca gland, sp. spermatheca.

**Figure 20.**
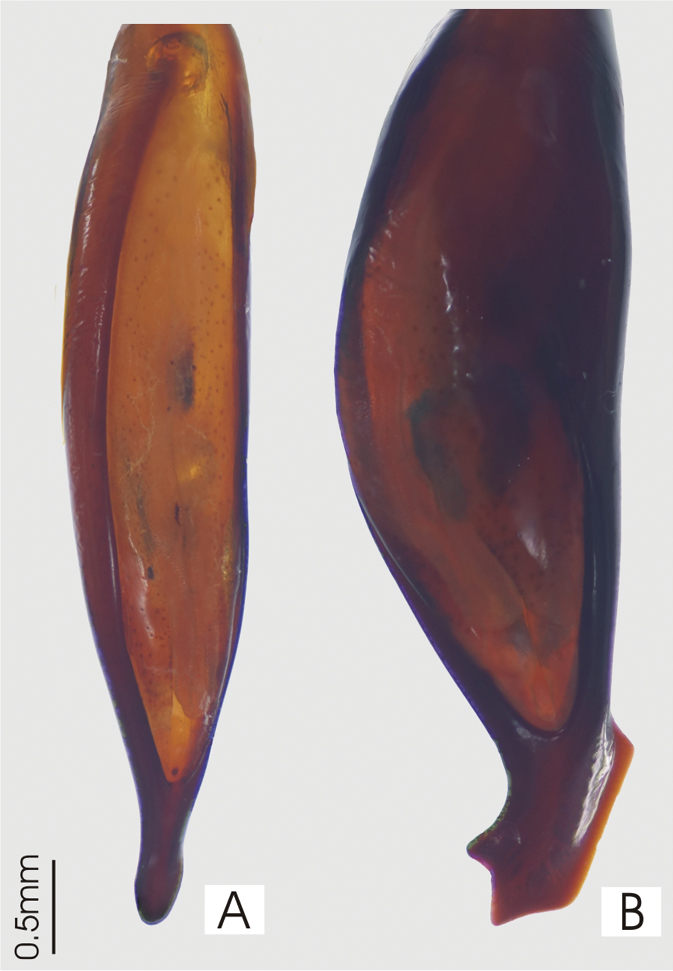
Male aedeagus, dorsal view of blade **A** *Abacoleptus curtus* **B** *Abacoleptus carinatus*.

*Head*. Clypeo-ocular sulci moderately or shallowly impressed, straight or very slightly divergent; mentum deeply emarginate, sides very slightly divergent, paramedial pits absent; median tooth bifid; paraglossae small, without elongate setae at apex; ligular sclerite with two seta on apical margin; maxillary palpifer with one basal seta; antennae filiform, with three basal segments glabrous. *Thorax*. Pronotum trapezoidal, widening basad, two marginal setae; prosternal process with four to six apical setae; mesosternum with two to four apical setae; metasterna glabrous; proepisternum smooth; elytra fused, bordered at base, striae 1-6 very shallowly impressed, stria 7 very shallowly impressed or absent, striae 8-9 impressed but scarcely separated from each other and somewhat difficult to discern, apicolateral plica large and visible, parascutellar stria very shallowly impressed, obscured or absent, angular base of stria 1 absent or rarely very shallowly impressed and obscured, parascutellar setigerous punctures absent, no discal punctures, interval 7 sharply carinate throughout, interval 5 sharply carinate or raised and rounded in apical third, other intervals flat or convex; hind wing reduced; anterior tarsi of male with three basal segments expanded and squamose beneath, all tarsi dorsally glabrous. *Abdomen*. Ventrites 3-6 without sulci; aedeagus (Figs 18, 20) ostium dorsal, median lobe oriented left side up in repose; parameres broad, attenuate, right with long apex, left with small denticle; female reproductive tract (fig. 19) without dorsolateral bursal lobe, elongate spermatheca broadly attached laterally at base of common oviduct, spermatheca with appended gland, spermatheca with digitiform diverticulum near base, without spermathecal gland duct diverticulum.

#### New Caledonian species.

*Abacoleptus carinatus* Fauvel 1903 [type IRSNB!]

*Abacoleptus paradoxus* Heller 1916 [type MTD!]

*Abacoleptus curtus*Will sp. n.

#### Exemplars of species examined.

 All three New Caledonian species.

#### Generic distribution.

 New Caledonian precinctive.

### Abacoleptus curtus

Will sp. n.

urn:lsid:zoobank.org:act:B5AFEE86-1AC3-4AB4-8254-CC8AC16A42F0

http://species-id.net/wiki/Abacoleptus_curtus

Figs 17C 20A

#### Type Locality.

 New Caledonia, Northern Province, Aoupinie, 21°11'S, 165°19'E.

#### Type material.

 Holotype, male (EMEC80970), “NEW CALEDONIA 8715, 21 11Sx165 19E, Aoupinie, top camp, 2-3Nov2001, C.Burwell, G.Monteith, hand collect”, deposited MNHN. Paratype, male (EMEC80971), same data as holotype [QM].

#### Description.

*Size*. Overall length (sbl) 21.3-21.8mm, greatest width over elytra 8.3-9.0mm. *Color*. Dorsal and ventral surfaces black, legs, mouthparts, and antennomeres black to piceous. *Luster*. Dorsal surface dull, head and lateral edges of elytra contrastingly smoother and shinier, ventral surfaces moderately shiny. *Iridescence*. No spectral iridescence dorsally. Ventral surface of body with slight spectral iridescence laterally on abdomen and on prothorax. *Head*. Dorsal microsculpture with microlines hardly visible at 50x magnification, sculpticells small, forming isodiametric or slightly irregular mesh, clypeal-ocular sulci not impressed, represented as broad shallow depressions, ocular ratio 1.3, eyes small, rounded, not prominent, post-ocular small. Labrum anterior margin straight. Antennae: Overall length very long, antennomeres 8-11 reaching beyond base of pronotum, antennomeres very elongate. *Thorax*. Pronotum trapezoidal, base notably wider than apex, sides shallowly rounded to base, marginal bead continuous from apex to near base, base without marginal bead, hind angle right angled, anterior margin emarginate, anterior angles produced, inner basal impressions broad, shallow, not well impressed or defined, convergent, outer impression lacking, seta at hind angle in basal marginal bead, about one pore width from lateral margin. Dorsal surface dull, microsculpture visible at 50x magnification as irregular mesh. Macrosculpture densely pappilous laterally, irregularly rugose on disc. Elytral striae complete, very shallowly impressed, smooth. Parascutellar stria present, angular base of stria 1 absent. Elytra dull except for contrastingly shiny intervals 8-9, microsculpture visible at 50x as irregular mesh. Macrosculpture densely pappilous. Metacoxal sulcus straight and ended at lateral end of coxa. *Abdomen*. Last abdominal ventrite with narrow apical bead. Female unknown, male aedeagus (20A) narrow, with tip narrow and slightly expanded and rounded.

Etymology. The specific epithet, *curtus* is Latin for short, in recognition of the relatively short and broad form of these beetles is treated as an adjective.

### Genus Platysmodes

Fauvel, 1903:231

http://species-id.net/wiki/Platysmodes

**Figure 21.**
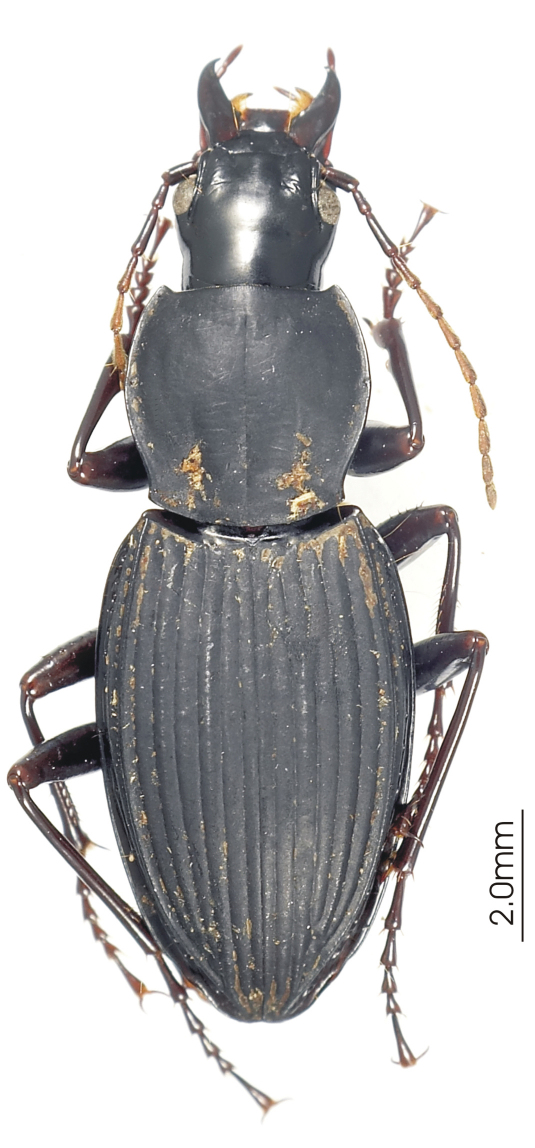
Habitus image, *Platysmodes gambeyi*.

Figs 21 [Fig F22] 23

#### Type species.

*Sphodrosomus gambeyi* Fauvel 1882, by monotypy.

#### Description.

**Figure 22. F22:**
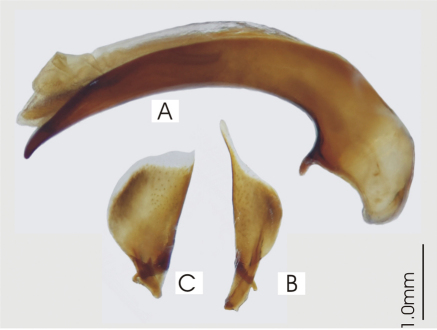
Male aedeagus of *Platysmodes gambeyi* **A** right lateral view **B** right paramere **C** left paramere.

**Figure 23.**
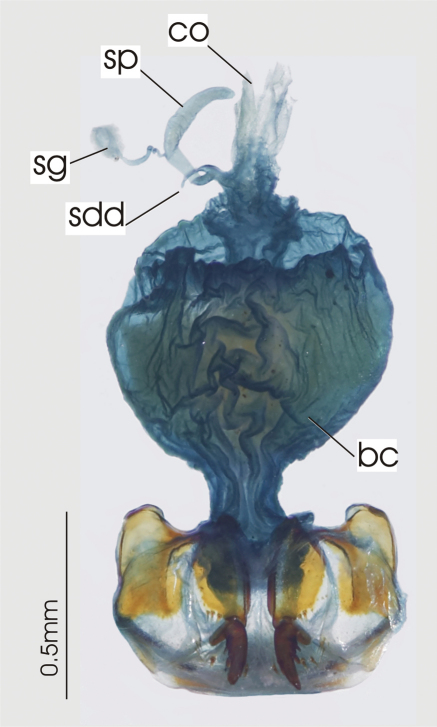
Female reproductive tract, ventral view. *Platysmodes gambeyi*, bc. bursa copulatrix, co. common oviduct, spermatheca digitiform diverticula sg. spermatheca gland, sp. spermatheca.

*Head*. Clypeo-ocular sulci long, well impressed, divergent; mentum emarginate, sides slightly divergent, paramedial pits moderate size, deeply impressed; median tooth bifid; paraglossae small, without elongate setae at apex; ligular sclerite with two seta on apical margin; maxillary palpifer with one basal seta; antennae filiform, with three basal segments glabrous. *Thorax*. Pronotum elongate cordiform, margins sinuate basad, two marginal setae; pro-, meso- and metasterna glabrous; proepisternum smooth; elytra fused, without distinct border medially at base, nine well impressed striae, very short tenth stria at level of plica, apicolateral plica large and visible, parascutellar stria short, very shallowly impressed, not connected to stria 1, angular base of stria 1 present, shallowly impressed, parascutellar punctures present at base of stria 2, no discal punctures, interval 7 sharply raised throughout its length, interval 8 slightly raised in the basal half, other intervals slightly convex becoming flatter apicad; hind wing reduced; anterior tarsi of male with three basal segments expanded and squamose beneath, all tarsi dorsally glabrous. *Abdomen*. Ventrites 3-6 without sulci; aedeagus (fig. 22) ostium dorsal, oriented left side up in repose; right paramere long, tip attenuate, left short, rounded with small membranous denticle at apex; female reproductive tract (fig. 23) without dorsolateral bursal lobe, elongate spermatheca broadly attached laterally at base of common oviduct, spermatheca with appended gland, spermatheca with digitiform diverticulum near base, without spermathecal gland duct diverticulum.

#### New Caledonian species.

*Platysmodes gambeyi* (Fauvel 1882) [type IRSNB!]

*=Sphodrosomus gambeyi* Fauvel 1882

#### Generic distribution.

 New Caledonian precinctive.

### Genus Cerabilia

Laport de Castelnau, 1867:116

http://species-id.net/wiki/Cerabilia

**Figure 24.**
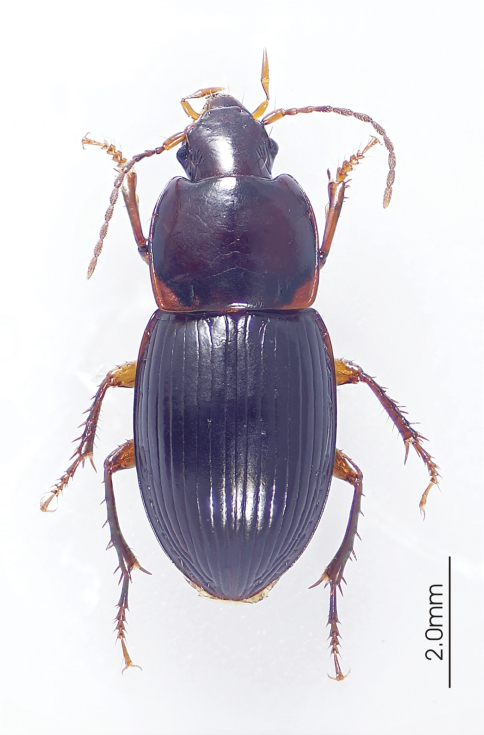
Habitus image, undescribed *Cerabilia* species “NC15”.

Figs 24 [Fig F25] 26

#### Type species.

*Cerabilia maori* Laport de Castelnau 1867, by monotypy.

#### Description.

*Head*. Clypeo-ocular sulci absent; mentum moderately emarginate, sides divergent, paramedial pits range from small and shallow to very large and deeply impressed; median tooth simple, triangular or blunt; paraglossae moderate length or short, without elongate setae at apex; ligular sclerite with two subapical setae; maxillary palpifer with one basal seta; antennae filiform, with first two segments always glabrous, pubescence starting in apical half of third segment to middle of fourth segment.

**Figure 25. F25:**
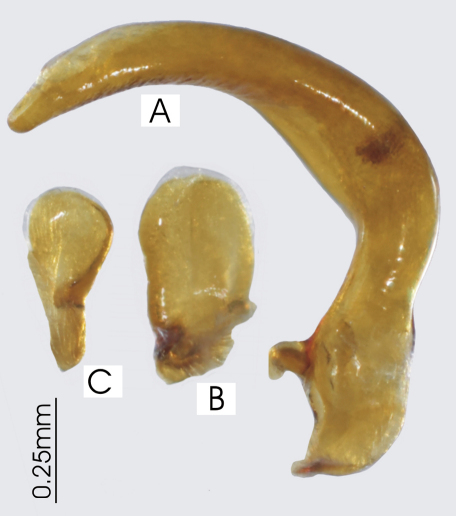
Male aedeagus of undescribed *Cerabilia* species “NC15” **A** right lateral view **B** right paramere **C** left paramere.

**Figure 26.**
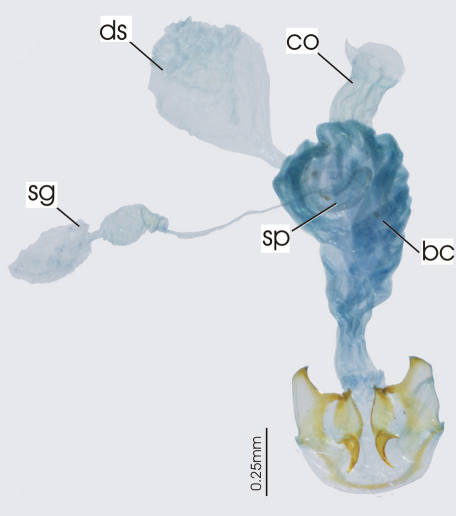
Female reproductive tract, ventral view. Undescribed *Cerabilia* species “NC15”, bc. bursa copulatrix, co. common oviduct, ds. dorsal sac of bursa, sg. spermatheca gland, sp. spermatheca.

*Thorax*. Pronotum transverse, one marginal setae near middle or with two marginal setae (two in Australian and some New Zealand species); pro-, meso- and metasterna glabrous; proepisternum smooth or deeply, longitudinally strigous; elytra fused, border at base, nine fully impressed striae, short tenth stria at level of plica in some species (some Australian species striae not impressed throughout or not impressed in basal fifth), apicolateral plica absent, parascutellar stria continuous with stria 1, angular base of stria 1 absent, parascutellar punctures present at base of stria 2, interval 3 with zero to five punctures (no discal punctures in most New Zealand and all Australian species), intervals flat or slightly convex; hind wing reduced; anterior tarsi of male with three basal segments, very slightly, somewhat or notably asymmetrically expanded, ventrally squamous, tarsi dorsally glabrous or with fine scattered setae. *Abdomen*. Ventrites 3-6 without transvers sulci; aedeagus (fig. 25) ostium dorsal, oriented left side or right side up in repose; right paramere small, bluntly rounded, left conchoid, paramere form reversed in species with adeagus right side up in repose; female reproductive tract (fig. 26) without dorsolateral bursal lobe, bursa with large dorsal sac present or absent, spermatheca small, sessile, broadly attached at base of common oviduct, spermatheca with appended gland, without spermatheca duct digital diverticulum, without elongate spermathecal gland duct diverticulum.

#### New Caledonian species.

*Cerabilia* includes approximately 24 undescribed species in New Caledonia. These will be described elsewhere.

#### Exemplars of species examined.

 All New Caledonian species, all Australian species (three described in *Feronista* and 23 undescribed) and all New Zealand species (five described and 10 undescribed).

#### Generic distribution.

 If considered congeneric with *Feronista* (see notes below), range is Australia, New Caledonia, New Zealand.

#### Notes.

*Cerabilia* has been treated as an endemic genus from New Zealand and has typically been classified in Platynini (Lorenz 2005a, b, Larochelle and Larivière 2007). Analysis of a combination of morphological characters and DNA data place *Cerabilia* in Loxandrini as sister to, or within a clade of Australian *Feronista* (Will unpubl.). Although like platynines *Cerabilia* species lack the lateroapical elytral plica, which is present in most loxandrines, they lack the dorsal lobe of the pygidial gland reservoir (present in species of Platynini), lack the angular base of stria 1 (usually present in species of Platynini) and males in *Cerabilia* have the three basal protarsomeres slightly, to very notably asymmetrically expanded, a characteristic of nearly all loxandrines. Tentatively, I consider species in New Caledonia to be included in *Cerabilia*. A full analysis of all Australian, New Zealand and New Caledonian species and presumed outgroups is to be published elsewhere establishing the evidentiary basis for a generic classification of Loxandrini.

### Genus Abacomorphus

Chaudoir, 1878:14

http://species-id.net/wiki/Abacomorphus

**Figure 27.**
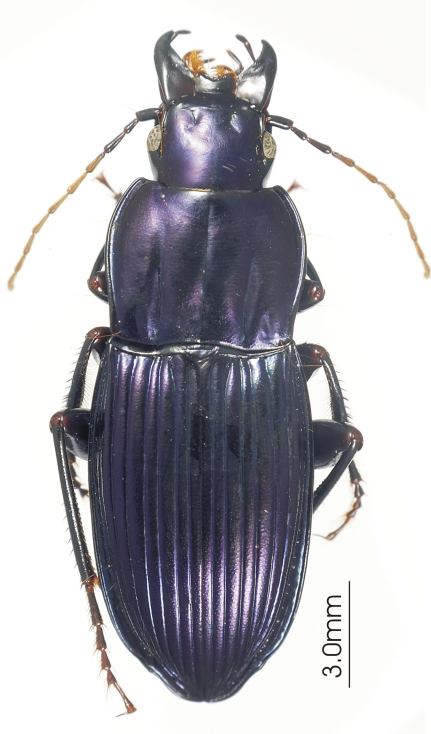
Habitus image, *Abacomorphus caledonicus*.

Figs 27 [Fig F28] 29

#### Type species.

*Abax caledonicus* Montrouzier 1860, by monotypy.

#### Description.

**Figure 28. F28:**
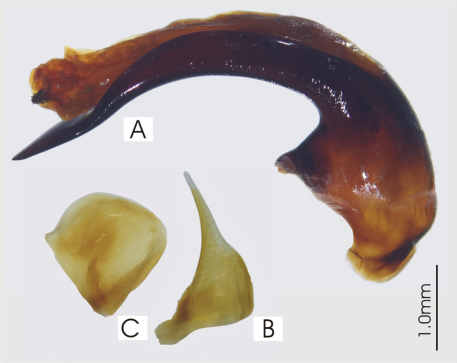
Male aedeagus of *Abacomorphus caledonicus* **A** right lateral view **B** right paramere **C** left paramere.

**Figure 29.**
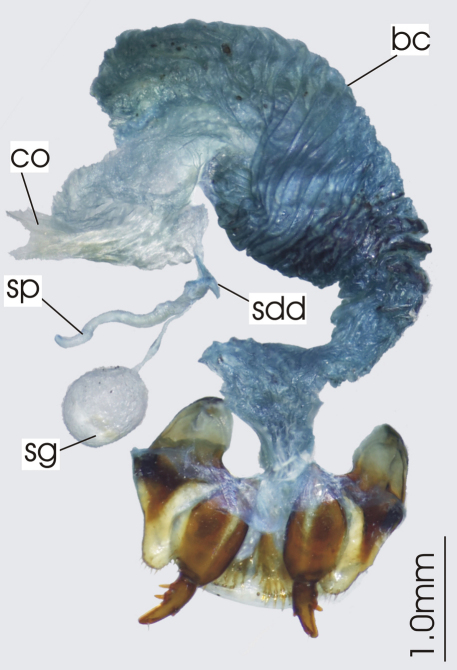
Female reproductive tract, ventral view. *Abacomorphus caledonicus*, bc. bursa copulatrix, co. commonoviduct, sdd. spermatheca digitiform diverticula, sg. spermatheca gland, sp. spermatheca.

*Head*. Clypeo-ocular sulci moderately or deeply impressed, nearly punctiform or sharply divergent; mentum moderately emarginate, sides divergent, paramedial pits small, clearly impressed; median tooth narrow, bifid; paraglossae long, without elongate setae at apex; ligular sclerite with two seta on apical margin; maxillary palpifer with one basal seta; antennae filiform, with three basal segments glabrous. *Thorax*. Pronotum quadrate, margins sinuate basad, two marginal setae; prosternal process with two to four apical setae; meso- and metasterna glabrous; proepisternum smooth; elytra fused, bordered at base, nine well impressed striae, some individuals with short, shallow tenth stria at level of plica, apicolateral plica large and visible, parascutellar stria well impressed, angular base of stria 1 absent, parascutellar setigerous punctures present at base of stria 2, no discal punctures, interval 7 sharply carinate near base, other intervals slightly or moderately convex; hind wing reduced; anterior tarsi of male with three basal segments expanded and squamose beneath, all tarsi dorsally glabrous or meso- and/or metatarsi with a few scattered setae. *Abdomen*. Ventrites 3-6 without sulci; aedeagus (fig. 28) ostium dorsal, median lobe oriented left side up in repose; parameres, right attenuate with long apex, left broad, rounded; female reproductive tract (fig. 29) without dorsolateral bursal lobe, elongate spermatheca broadly attached laterally at base of common oviduct, spermatheca with appended gland, spermatheca with digitiform diverticulum near base, without spermathecal gland duct diverticulum.

#### Exemplars of species examined.

*Abacomorphus asperulus* and *Abacoleptus caledonicus*.

#### Generic Distribution.

 New Caledonian precinctive.

#### New Caladonian species.

*Abacomorphus asperulus* Fauvel 1882 [type IRSNB!]

*Abacomorphus caledonicus* (Montrouzier 1860) [type MNHN]

*=Abax caledonicus* Montrouzier 1860

#### Notes.

 Among the more than twenty specimens of *Abacomorphus caledonicus* I examined there is significant variation in size, color and to a lesser degree microsculpturing. However, male genitalia were very uniform. This contrasts with *Abacoleptus asperulus*, which had little variation in size and color.

### Genus Abacophrastus gen n.

urn:lsid:zoobank.org:act:640DF22D-5256-4068-8E9E-70F24D2FE0BB

http://species-id.net/wiki/Abacophrastus

**Figure 30.**
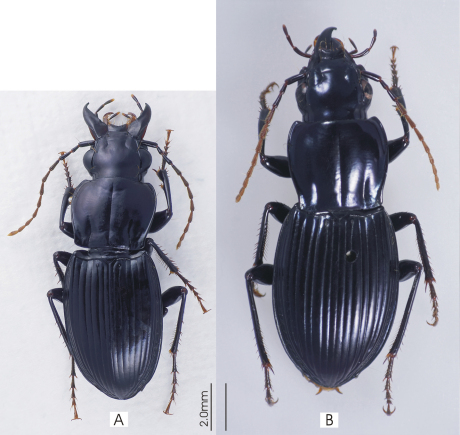
Habitus image **A** *Abacophrastus megalops* **B** *Abacophrastus bellorum*.

Figs 30 [Fig F31] [Fig F32] 33

#### Type species.

*Abacophrastus millei* Will sp. n.

#### Generic Description.

**Figure 31. F31:**
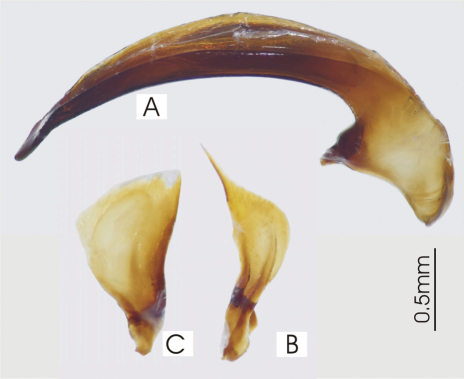
Male aedeagus of *Abacophrastus megalops* **A** right lateral view **B** right paramere **C** left paramere.

**Figure 32. F32:**
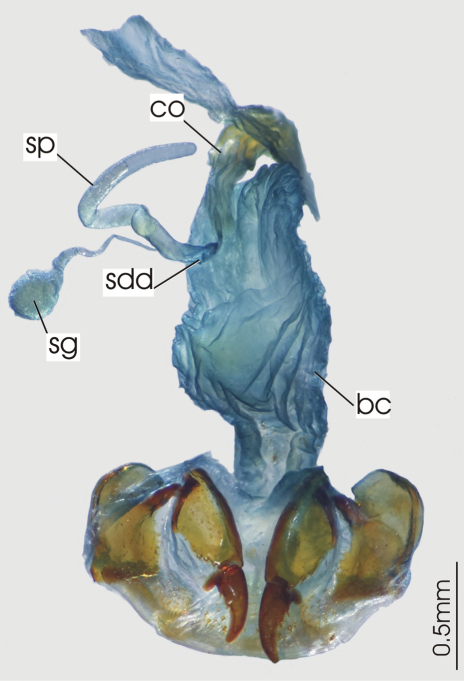
Female reproductive tract, ventral view. *Abacophrastus megalops*, bc. bursa copulatrix, co. common oviduct, sdd. spermatheca digitiform diverticula, sg. spermatheca gland, sp. spermatheca.

**Figure 33.**
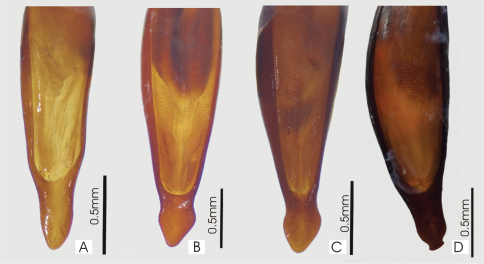
Male aedeagus, dorsal view of blade for A. *Abacophrastus millei* **B** *Abacoleptus hobbit* **C** *Abacoleptus megalops* **D** *Abacoleptus chapes*.

*Head*. Clypeo-ocular sulci moderately to deeply impressed, divergent; mentum moderately emarginate, sides divergent, paramedial pits small, clearly impressed; median tooth bifid; paraglossae small, without elongate setae at apex; ligular sclerite with two seta on apical margin; maxillary palpifer with one basal seta; antennae filiform, with three basal segments glabrous. *Thorax*. Pronotum quadrate, margins sinuate basad, two marginal setae; pro-, meso- and metasterna glabrous; proepisternum smooth; elytra fused, bordered at base, nine well impressed striae, some individuals with a very short tenth stria at level of plica, apicolateral plica large and visible, parascutellar stria well impressed, angular base of stria 1 absent, parascutellar setigerous punctures present at base of stria 2, no discal punctures, humeri prominent, interval 7 slightly or notably raised near base, slightly more convex than other intervals throughout its length, other intervals slightly or moderately convex; hind wing reduced; anterior tarsi of male with three basal segments expanded and squamose beneath, all tarsi dorsally with scattered long setae. *Abdomen*. Ventrites 3-6 without sulci; aedeagus (Figs 31, 33) ostium dorsal, oriented left side up in repose; right paramere long, tip attenuate, left short, broader, narrowly rounded at apex; female reproductive tract (fig. 32) without dorsolateral bursal lobe, elongate spermatheca broadly attached laterally at base of common oviduct, spermatheca with appended gland, spermatheca with very small digitiform diverticulum near base, without spermathecal gland duct diverticulum.

#### Etymology.

 The genus is treated as Latin masculine and is a combination incorporating the Greek *abaco*, being broad and therefore like the genus *Abax*, and *phrastos* meaning “expression”. Given that these species are similar and related to *Abacomorphus* and *Abacoleptus*, the name roughly translates as yet “another expression of *Abax*.”

#### New Caldonian species.

*Abacophrastus millei* Will sp. n.

*Abacophrastus bellorum* Will sp. n.

*Abacophrastus carnifex* Will sp. n.

*Abacophrastus chapes* Will sp. n.

*Abacophrastus hobbit* Will sp. n.

*Abacophrastus megalops* Will sp. n.

*Abacophrastus reflexus* Will sp. n.

### Abacophrastus millei

Will sp. n.

urn:lsid:zoobank.org:act:EC9488D5-3B66-4C0E-A53A-25443741883D

http://species-id.net/wiki/Abacophrastus_millei

Fig. 33A

#### Type locality.

 New Caledonia, Southern Province, Col d’Amieu 21°34'29"S, 165°45'19"E, 510m.

#### Type material.

 Holotype, male (EMEC80965), “21°34'29"S/165°45'19"E, NEW CALEDONIA:Prov. Sud, Col d’Amieu, 510m. 16.iii.2007 K.Will, leaf litter, logs”, deposited MNHN. Paratype, “21°35'11"S/165°46'30"E, NEW CALEDONIA:Prov. Sud, Col d’Amieu, 485m. 14.iii.2007 K.Will, ex leaf litter”, female (EMEC80962) (teneral) [EMEC].

#### Description.

*Size*. Overall length (sbl) 9.0-9.1mm, greatest width over elytra 3.4-3.5mm.

*Color*. Dorsal and ventral surfaces black, legs, mouthparts, and antennae very slightly paler than ventral surface of body, piceous. *Luster*. Dorsal and ventral surfaces shiny.

*Iridescence*. Elytra and ventral surface of body with moderate spectral iridescence.

*Head*. Dorsal microsculpture with microlines visible at 50x magnification, sculpticells isodiametric or slightly irregular, forming mesh, clypeal-ocular sulci very broad and scarcely impressed, ocular ratio 1.5-1.6, eyes average size for genus, rounded flat, not prominent, post-ocular orbits large. Labrum broadly emarginate. Antennae: Overall length moderately long, just reaching beyond base of pronotum, antennomeres 5-11 moderately elongate. *Thorax*. Pronotum nearly quadrate, sides evenly rounded in apical 3/4 and then sinuate and straight to base, marginal bead continuous from apex to sinuation then broadening and not delimited medially, hind angle slightly explanate, rounded but nearly right-angled, anterior margin slightly emarginate, anterior angles moderately produced, inner basal impressions linear, slightly divergent and well impressed, outer impression lacking, seta at hind angle well forward of basal margin, about half length of straight portion of margin and nearly as distant from lateral margin. Dorsal surface shiny, microsculpture scarcely visible as irregular mesh of microlines at 50x magnification. Elytral striae complete, well impressed and impunctate. Elytra shiny, microsculpture readily visible at 50x as stretched mesh of microlines. Metacoxal sulcus straight and ended well before lateral end of coxa. *Abdomen*. Last abdominal ventrite with narrow, light apical bead. Male aedeagus tip elongate with simply rounded point.

#### Etymology.

 The specific epithet is a noun in the genitive that honors Christian Mille of the Institut Agronomique néo-Calédonien.

### Abacophrastus chapes

Will sp. n.

urn:lsid:zoobank.org:act:0FB19F1F-6A0E-47FA-9BF1-6DC136476A3C

http://species-id.net/wiki/Abacophrastus_chapes

Fig. 33D

#### Type locality.

 New Caledonia, Northern Province, Poindimié, Village de Napoémien.

#### Type material.

 Holotype, male (EMEC10003808), “NEW CALEDONIA Poindimie 100m V.of Nepoemien 14 Jan 1979 A.Renevier”, deposited MNHN.

#### Description.

*Size*. Overall length (sbl) 13.6 mm, greatest width over elytra 5.1mm.

*Color*. Dorsal and ventral surfaces black with a distinct blue reflex, legs, mouthparts, and antennae black, concolorous with ventral surface of body. *Luster*. Dorsal and ventral surfaces shiny. *Iridescence*. Elytra and ventral surface of body without spectral iridescence. *Head*. Dorsal microsculpture with microlines scarcely visible at 50x magnification, sculpticells slightly irregular, forming mesh, clypeal-ocular sulci very short, well impressed, ocular ratio 1.5, eyes average size for genus, rounded moderately prominent, post-ocular orbits large. Labrum broadly emarginate. Antennae: Type is damaged, with only 4 basal antenomeres so length unknown. *Thorax*. Pronotum nearly quadrate, sides scarcely rounded, with only a very slight sinuation near base, marginal bead continuous from apex to near hind angle, hind angle scarcely explanate, rounded and slightly obtuse angle, anterior margin slightly emarginate, anterior angles moderately produced, inner basal impressions linear, slightly divergent and well impressed, outer impression lacking, seta at hind angle well forward of basal margin, about half length of straight portion of margin and nearly as distant from lateral margin. Dorsal surface shiny, microsculpture scarcely visible as irregular mesh of microlines at 50x magnification. Elytral striae complete, well impressed and impunctate. Elytra shiny, microsculpture hardly visible at 50x as stretched mesh of microlines. Metacoxal sulcus straight and ended well before lateral end of coxa. *Abdomen*. Last abdominal ventrite with narrow, light apical bead. Male aedeagus tip elongate with subapical constriction and dorso-ventrally thin, flat point.

#### Etymology.

 The specific epithet, *chapes*, is the terms for the rounded metal tip of the scabbard for a sword and refers to the form of the tip of the male adeagus in this species. It is treated as a noun in the nominative singular standing in apposition.

### Abacophrastus carnifex

Will sp. n.

urn:lsid:zoobank.org:act:4D4B1E11-61FB-4391-9EC7-C0E707970FB1

http://species-id.net/wiki/Abacophrastus_carnifex

#### Type locality.

 New Caledonia, Southern Province, Mt. Humboldt, 1330m.

#### Type material.

 Holotype, female (EMEC80966), “Nouvelle Calédonie m 1330. M. Humboldt for. Mousse 15.II.2006, P.M. Giachino leg.”, deposited MNHN.

#### Description.

*Size*. Overall length (sbl) 11.9mm, greatest width over elytra 4.7mm.

*Color*. Dorsal and ventral surfaces black, legs, mouthparts, and antennae black to piceous. *Luster*. Dorsal and ventral surfaces shiny. *Iridescence*. Elytra and ventral surface of body with slight spectral iridescence. *Head*. Dorsal microsculpture with microlines visible at 50x magnification, sculpticells isodiametric or slightly irregular, forming mesh, clypeal-ocular sulci impressed, short, linear, ocular ratio 1.4, eyes average size for genus, rounded moderately prominent, post-ocular orbits large. Labrum broadly emarginate. Antennae: Overall length long, antennomeres 10-11 reaching beyond base of pronotum, antennomeres 5-11 elongate. *Thorax*. Pronotum nearly quadrate, sides evenly rounded in apical 3/4 and then sinuate and straight to base, marginal bead continuous from apex to sinuation then broadening and not delimited medially, hind angle slightly explanate, rounded but nearly right-angled, anterior margin slightly emarginate, anterior angles moderately produced, inner basal impressions linear, slightly divergent and well impressed, outer impression lacking, seta at hind angle well forward of basal margin, about half length of straight portion of margin and nearly as distant from lateral margin. Dorsal surface shiny, microsculpture hardly visible as irregular mesh of microlines at 50x magnification. Elytral striae complete, impunctate, shallowly impressed, except laterally and apically. Stria 6 evanescent near base, not reaching basal margin. Elytra moderately shiny, microsculpture scarcely visible at 50x as stretched mesh of microlines. Metacoxal sulcus straight and ended well before lateral end of coxa. *Abdomen*. Last abdominal ventrite with narrow, light apical bead. Female tract not studied, male unknown.

#### Etymology.

 The specific epithet is *carnifex*, Latin for the “executioner” and is treated as a noun in the nominative singular standing in apposition.

### Abacophrastus hobbit

Will sp. n.

urn:lsid:zoobank.org:act:23AE1252-5169-47EE-A443-038ACFB87ADA

http://species-id.net/wiki/Abacophrastus_hobbit

Fig. 33B

#### Type locality.

 New Caledonia, Northern Province, Aoupinie, 850m, 21°11'S, 165°19'E.

#### Type material.

 Holotype, male (EMEC80967), “NEW CALEDONIA, 21°11'Sx165°19'E, Aoupinie, 850m, 9930, 20-21Nov2000, Bouchard, Burwell & Monteith", deposited MNHN. Paratypes, “NEW CALEDONIA, Aoupinié, 20km NE Poya, 650m, 18-19 May, 1984, G. Monteith & D. Cook", 2 females (EMEC10003809, EMEC10003810) [QM]. Pic d'Amoa, N. slopes, 20 58 Sx165 17 E, 10-11Nov2001, hand col. C. Burwell & g. Monteith, 8687, 1 female (EMEC10003811) [QM].

#### Description.

*Size*. Overall length (sbl) 13.1mm, greatest width over elytra 5.2mm. *Color*. Dorsal and ventral surfaces black, legs, mouthparts, and antennae black to piceous. Dorsally with distinct metallic purple reflex, more evident on head and pronotum. *Luster*. Dorsal surface very shiny, ventral surface moderately shiny. *Iridescence*. Elytra and base of pronotum with distinct spectral iridescence. Ventral surface of body with slight spectral iridescence. *Head*. Dorsal microsculpture with microlines visible at 50x magnification, sculpticells isodiametric or slightly irregular, forming mesh, clypeal-ocular sulci impressed, short, divergent, arcuate, ocular ratio 1.4, eyes average size for genus, rounded, moderately prominent, post-ocular orbits large. Labrum broadly emarginate. Antennae: Overall length long, antennomeres 10-11 reaching beyond base of pronotum, antennomeres 5-11 elongate. *Thorax*. Pronotum quadrate, sides nearly straight, slightly rounded in apical 3/4 and then sinuate and straight to base, marginal bead continuous from apex to near base, broadening slightly near base, hind angle slightly explanate, nearly right-angled, anterior margin slightly emarginate, anterior angles prominently produced, inner basal impressions linear and well impressed, outer impression lacking, seta at hind angle forward of basal margin, about one third length of straight portion of margin and as distant from lateral margin. Dorsal surface shiny, microsculpture hardly visible as irregular mesh of microlines at 50x magnification. Elytral striae complete, well impressed except stria 6, impunctate. Stria 6 evanescent in basal third, not reaching basal margin. Elytra shiny, microsculpture scarcely visible at 50x as stretched mesh of microlines. Metacoxal sulcus straight and ended well before lateral end of coxa. *Abdomen*. Last abdominal ventrite with narrow, light apical bead. Female unknown, male aedeagus (fig. 33B) tip bluntly and narrowly deltoid.

#### Etymology.

 The specific epithet Hobbit is an allusion to the setose dorsal surface of the tarsi, analogous to the hairy feet of Tolkien’s Hobbits and is treated as a noun in the nominative singular standing in apposition.

### Abacophrastus megalops

Will sp. n.

urn:lsid:zoobank.org:act:C31CF376-7F63-4A91-8122-A61094ED7150

http://species-id.net/wiki/Abacophrastus_megalops

Figs 30 [Fig F31] 32 33C

#### Type locality.

 New Caledonia, Southern Province, Mt. Koghis, 700m, 22°10'28"S, 166°30'48"E.

#### Type material.

 Holotype, female (EMEC80964), “22°10'28"S/166°30'48"E, NEW CALEDONIA, Prov. Sud, Mt. Koghis, 700m el. Coll. K.Will, 12.iii.2007, headlamp search”, deposited MNHN. Paratypes, same data as holotype, 1 female (teneral), 1 male (EMEC80963) (disarticulated, stored in EtOH) [EMEC]

#### Description.

*Size*. Overall length (sbl) 10.6-11.0mm, greatest width over elytra 4.0-4.2mm. *Color*. Dorsal and ventral surfaces black, legs, mouthparts, and antennomeres 1-5 black to piceous, antennomeres 6-11 paler, slightly infuscated. Dorsally with very slight metallic purple reflex, more evident on head and pronotum. *Luster*. Dorsal surface very shiny, ventral surface moderately shiny. *Iridescence*. Elytra with distinct spectral iridescence. Ventral surface of body with slight spectral iridescence.

*Head*. Dorsal microsculpture with microlines visible at 50x magnification, sculpticells isodiametric or slightly irregular, forming mesh, clypeal-ocular sulci impressed, short, divergent, arcuate, ocular ratio 1.5-1.6, eyes large for genus, rounded, moderately prominent, post-ocular orbits relatively small. Labrum broadly emarginate. Antennae: Overall length long, antennomeres 10-11 reaching beyond base of pronotum, antennomeres 5-11 elongate. *Thorax*. Pronotum quadrate, sides shallowly rounded in apical 3/4 and then slightly sinuate and straight to base, marginal bead continuous from apex to near base there ended at level of seta, hind angle scarcely explanate, rounded, but about right angled, anterior margin slightly emarginate, anterior angles moderately produced, inner basal impressions linear, well impressed, slightly divergent, outer impression lacking, seta at hind angle well forward of basal margin, about one half length of straight portion of margin and about 3/4 this length distant from lateral margin. Dorsal surface shiny, microsculpture of irregular mesh of microlines hardly visible at 50x magnification. Elytral striae complete, impunctate, shallowly impressed except laterally and apically. Elytra shiny, microsculpture scarcely visible at 50x as stretched mesh of microlines. Metacoxal sulcus straight and ended well before lateral end of coxa. *Abdomen*. Last abdominal ventrite with narrow, light apical bead. Male aedeagus (Figs 31, 33C) tip roundly and narrowly deltoid.

#### Etymology.

 The specific epithet is a compound word from the Greek, *megas* (large) and *ops* (eyes), referring to the large eyes in this species. It is treated as an adjective in the nominative singular.

### Abacophrastus bellorum

Will sp. n.

urn:lsid:zoobank.org:act:2861B458-814A-4AE9-BFFF-FA08F8A5E6F2

http://species-id.net/wiki/Abacophrastus_bellorum

Fig. 30B

#### Type locality.

 New Caledonia, Northern Province, Mt. Taom summit, 960m, 20°47'S, 164°35'E.

#### Type material.

 Holotype, female (EMEC200674), “NEW CALEDONIA 11965, 20°47'Sx164°35'E, 960m, Mt. Taom summit, Site 3, 7Dec2004, G.Monteith, dung pitfall, rainforest”, deposited MNHN. Paratype, same data as holotype, 1 female (EMEC200675) [QM].

#### Description.

*Size*. Overall length (sbl) 13.8-14.3mm, greatest width over elytra 5.3mm. *Color*. Dorsal and ventral surfaces black, legs, mouthparts, and antennomeres 1-4 black to piceous, 5 infuscated, antennomeres 6-11 paler. Dorsally with very slight metallic purple reflex, more evident on head and pronotum. *Luster*. Dorsal surface shiny, ventral surface slightly duller. *Iridescence*. Elytra with slight spectral iridescence. Ventral surface of body with very slight spectral iridescence. *Head*. Dorsal microsculpture with microlines scarcely visible at 50x magnification, sculpticells isodiametric or slightly irregular, forming mesh, clypeal-ocular sulci impressed, short, divergent, arcuate, ocular ratio 1.44, eyes typical for genus, rounded, moderately prominent, post-ocular orbits relatively small. Labrum broadly emarginate. Antennae: Overall length long, antennomeres 10-11 reaching beyond base of pronotum, antennomeres 5-11 elongate. *Thorax*. Pronotum narrow quadrate, sides shallowly rounded in apical 3/4 and then clearly sinuate and straight to base, marginal bead continuous from apex to near base there ended at level of seta, hind angle explanate, rounded, but about right angled, anterior margin emarginate, anterior angles moderately produced, inner basal impressions linear, well impressed, slightly divergent, outer impression lacking, seta near hind angle well forward of basal margin, about one half length of straight portion of margin and about 3/4 this length distant from lateral margin. Dorsal surface shiny, microsculpture of irregular mesh of microlines hardly visible at 50x magnification. Elytral striae complete, impunctate, well impressed. Elytra shiny, microsculpture scarcely visible at 50x as stretched mesh of microlines. Metacoxal sulcus straight and ended well before lateral end of coxa. *Abdomen*. Last abdominal ventrite with narrow, light apical bead. Male unknown.

#### Etymology.

 The specific epithet, a noun in the genitive case, honors Ross and Joyce Bell and their exceptional contribution to entomology and the study of Carabidae.

### Abacophrastus reflexus

Will sp. n.

urn:lsid:zoobank.org:act:BDBDBBD1-5C6F-4F64-A399-EA0FC298E109

http://species-id.net/wiki/Euryabax

#### Type Locality.

 New Caledonia, Northern Province, Me Maoya camp, 1150m, 21°22'S, 165°20'E.

#### Type material.

 Holotype, female (EMEC200676), “NEW CALEDONIA 11161, 21°22'Sx165°20'E, Me Maoya camp, 1150m, 11-13Nov 2002, hand coll. Burwell, Monteith & Wright”, deposited MNHN.

#### Description.

*Size*. Overall length (sbl) 13.7mm, greatest width over elytra 5.4mm. *Color*. Dorsal and ventral surfaces black, legs, mouthparts, and antennomeres 1-3 black to piceous, antennomeres 4-11 paler, slightly infuscated. Dorsally with slight metallic purple reflex. *Luster*. Dorsal surface shiny, ventral surface slightly duller. *Iridescence*. Elytra with spectral iridescence. Ventral surface of body without spectral iridescence except slightly so on proepisternum. *Head*. Dorsal microsculpture with scarcely microlines visible at 50x magnification, sculpticells isodiametric or slightly irregular, forming mesh, clypeal-ocular sulci impressed, short, linear, divergent, ocular ratio 1.42, eyes typical for genus, rounded, moderately prominent, post-ocular orbits relatively small. Labrum broadly emarginate. Antennae: Overall length long, antennomeres 10-11 reaching beyond base of pronotum, antennomeres 5-11 elongate. *Thorax*. Pronotum quadrate, sides shallowly rounded in apical 3/4 and then slightly sinuate to base, marginal bead continuous from apex to near base there ended at level of seta, margin at hind angle broadly explanate, angle about right angled, anterior margin emarginate, anterior angles moderately produced, inner basal impressions linear, impressed, slightly divergent, outer impression lacking, seta near hind angle well forward of basal margin, about length of very straight straight portion of margin and the distant from lateral margin. Dorsal surface moderately shiny, microsculpture of irregular mesh of microlines hardly visible at 50x magnification. Elytral striae complete, impunctate, well impressed. Elytra shiny, microsculpture scarcely visible at 50x as stretched mesh of microlines. Metacoxal sulcus straight and ended well before lateral end of coxa. *Abdomen*. Last abdominal ventrite with narrow, light apical bead. Male unknown.

#### Etymology.

 The specific epithet draws attention to the broadly reflexed basal third of the pronotum and is treated as an adjective in the nominative singular.

### Genus Euryabax

Fauvel, 1903:230

http://species-id.net/wiki/Euryabax

Figs 34 35

#### Type species.

*Euryabax colossus* Fauvel 1903 by monotypy.

#### Description.

*Head*. Clypeo-ocular sulci broadly impressed; mentum moderately emarginate, sides divergent, paramedial pits absent; median tooth bifid; paraglossae small, without elongate setae at apex; ligular sclerite with two seta on apical margin; maxillary palpifer with one basal seta; antennae filiform, with three basal segments glabrous. *Thorax*. Pronotum trapezoidal, widening basad, two marginal setae; pro-, meso- and metasterna glabrous; proepisternum smooth; elytra fused, bordered at base, nine well impressed striae, very short tenth stria at level of plica, apicolateral plica large and visible, parascutellar stria well impressed, angular base of stria 1 absent, parascutellar setigerous punctures present at base of stria 2, no discal punctures, interval 7 notably raised near base, slightly more convex than other intervals throughout its length, other intervals convex; hind wing reduced; anterior tarsi of male with segments not expanded without ventral setae all tarsi dorsally glabrous. *Abdomen*. Ventrites 3-6 without sulci; aedeagus (fig. 35) ostium dorsal, oriented left side up in repose; right paramere long, tip attenuate, left broader with membranous denticle at apex; female unknown.

**Figure 34.**
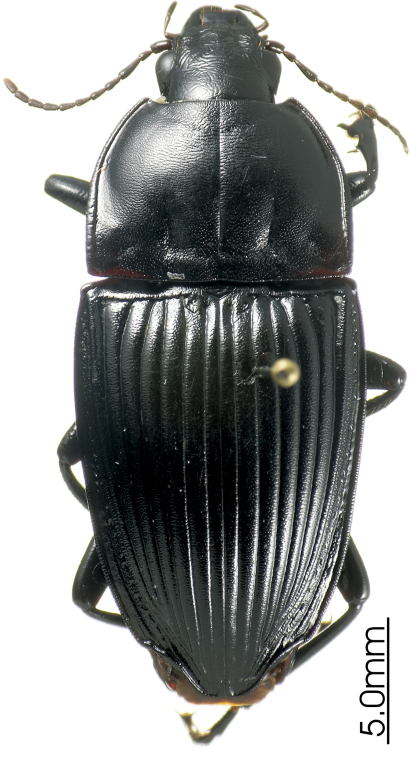
Habitus image, *Euryabax colossus*, holotype.

**Figure 35.**
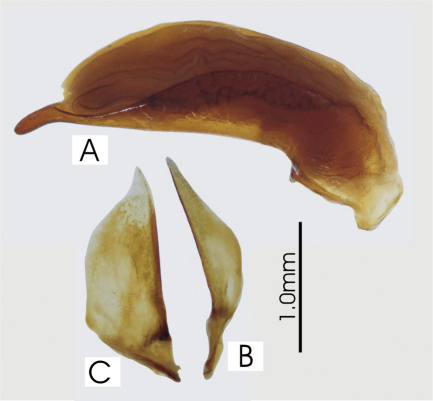
Male aedeagus of *Euryabax colossus*, holotype **A** right lateral view **B** right paramere **C** left paramere.

#### Generic Distribution.

 New Caledonian precinctive known only from type specimen.

#### New Caladonian species.

*Euryabax colossus* Fauvel 1903 [type IRSNB!]

### Genus Setalidius

Chaudoir, 1878:18

http://species-id.net/wiki/Setalidius

Fig. 36

#### Type species.

*Setalidius nigerrimus* Chaudoir 1878 by monotypy.

#### Description.

*Head*. Clypeo-ocular sulci shallow, not reaching anterior supraorbital seta or nearly absent, divergent; mentum only very slightly emarginate, sides widely divergent, epilobes not produced, paramedial pits very small, deeply impressed; median tooth bifid; paraglossae long, without elongate setae at apex; ligular sclerite with two seta on apical margin; maxillary palpifer with one basal seta; antennae filiform, with three basal segments glabrous. *Thorax*. Pronotum quadrate, slightly wider than long, l/w in *Setalidius nigerrimus* 0.88 and in *Setalidius attenuatus* 0.86 (contra Fauvel 1882b), margins very slightly sinuate to base, two marginal setae; pro-, meso- and metasterna glabrous; proepisternum smooth; elytra fused, border at base, nine fully impressed striae, very short tenth stria at level of plica, apicolateral plica large and visible, parascutellar stria short, shallowly impressed, not continuous with stria 1, angular base of stria 1 absent, parascutellar punctures absent, no discal punctures, intervals convex; hind wing reduced all tarsi dorsally glabrous. *Abdomen*. Ventrites 3-6 without transverse sulci; male unknown, female reproductive tract not studied.

**Figure 36.**
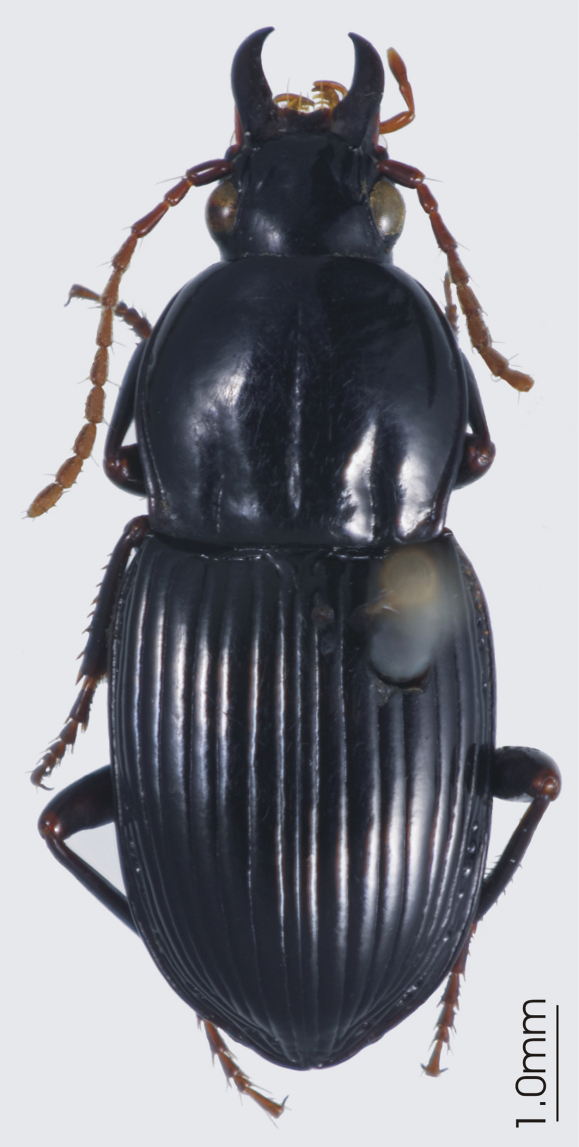
Habitus image, *Setalidius attenuatus*, holotype.

#### Exemplars of species examined.

 Both species, only known from the holotypes.

#### Generic Distribution.

 New Caledonian precinctive.

#### New Caladonian species.

*Setalidius nigerrimus* Chaudoir 1878 [type MNHN!]

*Setalidius attenuatus* Fauvel 1882 [type IRSNB!]

### Genus Sphodrosomus

Perroud, in Perroud and Montrouzier, 1864:58

http://species-id.net/wiki/Sphodrosomus

**Figure 37.**
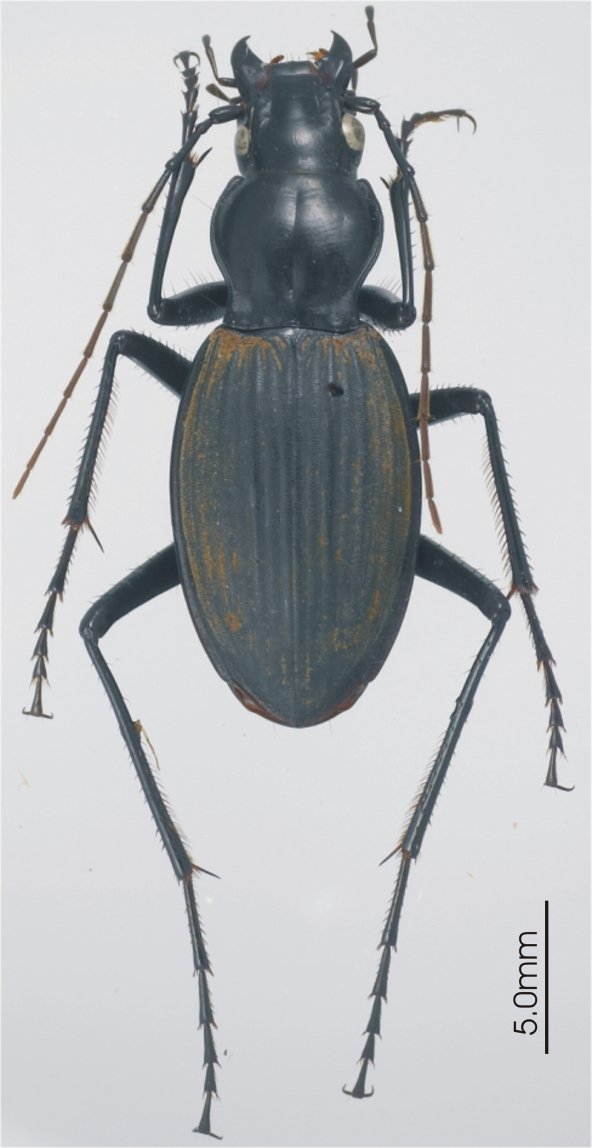
Habitus image, *Sphodrosomus saisseti*.

Figs 37 [Fig F38] [Fig F39] [Fig F40] [Fig F41] 42 43B

#### Type species.

*Sphodrosomus saisseti* Perroud and Montrouzier 1864, by monotypy.

#### Description.

**Figure 38. F38:**
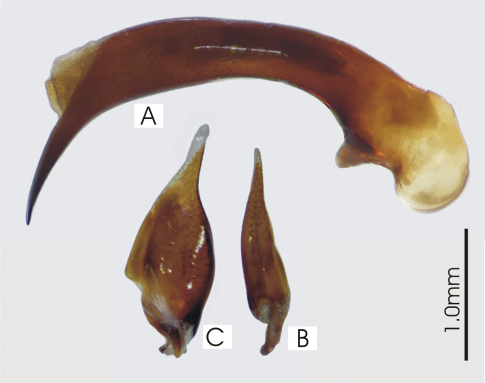
Male aedeagus of *Sphodrosomus saisseti* **A** right lateral view **B** right paramere **C** left paramere.

**Figure 39. F39:**
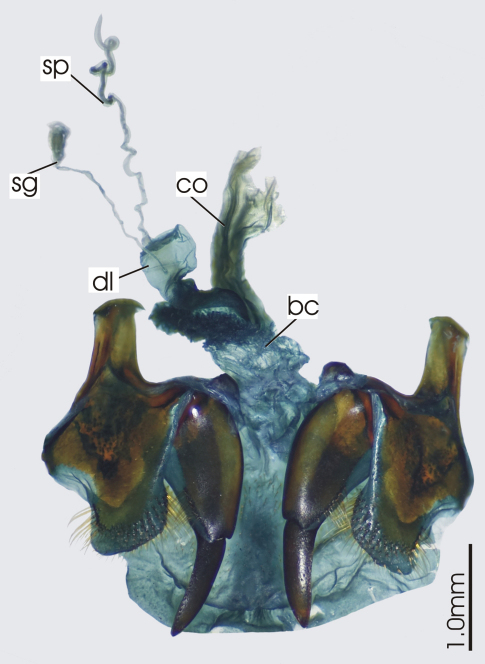
Female reproductive tract, ventral view. *Sphodrosomus saisseti*, bc. bursa copulatrix, co. common oviduct, dl. dorsal lobe of the bursa, sg. spermatheca gland, sp. spermatheca.

*Head*. Clypeo-ocular sulci long, straight, very broadly and very shallowly impressed; mentum emarginate, sides slightly divergent, paramedial pits absent; median tooth bifid; paraglossae small, without elongate setae at apex; ligular sclerite with two or four seta on apical margin; maxillary palpifer with one basal seta; antennae filiform, with three basal segments glabrous. *Thorax*. Pronotum cordiform, margins sinuate basad, two marginal setae; pro-, meso- and metasterna glabrous; proepisternum smooth; elytra fused, border at base, nine well impressed striae, with or without very short tenth stria at level of plica, apicolateral plica large and visible, parascutellar stria short, impressed, not connected to stria 1, angular base of stria 1 well impressed, parascutellar punctures absent or if present minute and present at base of stria 2, no discal punctures, interval 7 equally convex as other intervals or slightly raised near base, odd numbered intervals slightly raised and more convex or equal, all intervals becoming flatter apicad; hind wing reduced; anterior tarsi of male with three basal segments expanded and squamose beneath in *Setalidius griseolum*, segments not expanded without ventral setae in other species, all tarsi dorsally glabrous. *Abdomen*. Ventrites 3-6 without sulci; aedeagus (Figs 38, 41) ostium dorsal, median lobe oriented left side up in repose; parameres very attenuate with long apex, nearly of equal length; female reproductive tract (Figs 39, 42) with prominent dorsolateral bursal lobe, elongate spermatheca attached apically on lobe, accessory gland attached to bursa at base of lobe, without spermathecal duct digital diverticulum, without spermathecal gland duct diverticulum.

**Figure 40. F40:**
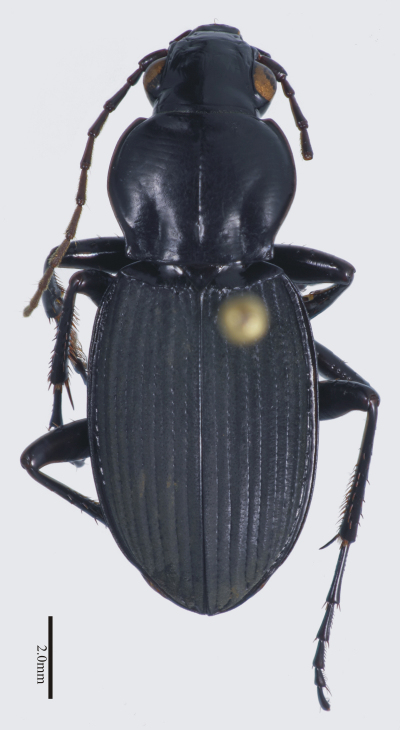
Habitus image, *Sphodrosomus griseolum*.

**Figure 41. F41:**
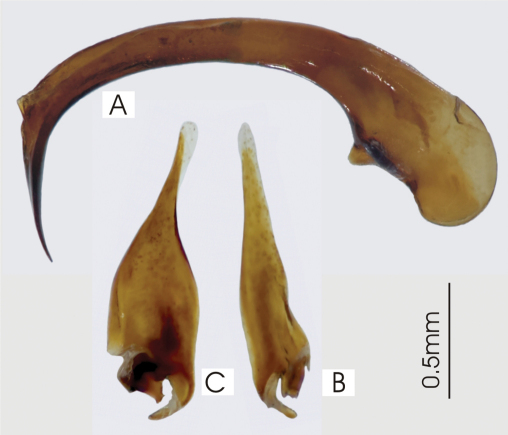
Male aedeagus of *Sphodrosomus griseolum* **A** right lateral view **B** right paramere **C** left paramere.

**Figure 42.**
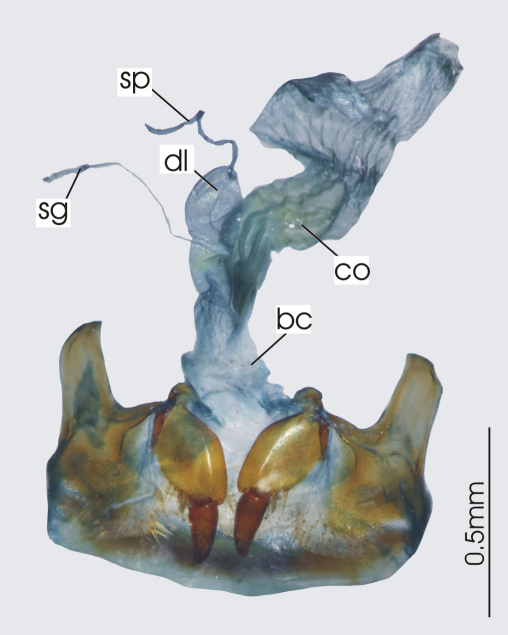
Female reproductive tract, ventral view. *Sphodrosomus griseolum*, bc. bursa copulatrix, co. common oviduct, dl. dorsal lobe of the bursa, sg. spermatheca gland, sp. spermatheca.

**Figure 43.**
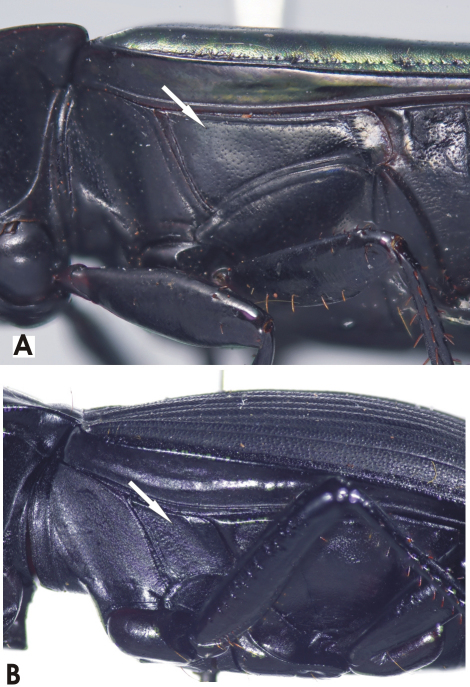
Left lateral view of metathorax showing **A** elongate metepisternum in *Platycaelus melliei* **B** short metepisternum in *Sphodrosomus saisseti*.

#### Exemplars of species examined.

 See treatment by Will (2006) for *Setalidius saisseti* and *Setalidius monteithi*. I examined four specimens in the Fauvel collection in IRSNB initially thought to be possible syntypes of *Homalosoma griseolum* Fauvel. The specimens are consistent with the morphological attributes as in the original description. However, in the original description Fauvel stated that the single specimen was from “Ile des Pins (Bougier)” a unique from the “Collection Gambey.” Subsequently, Fauvel (1903) published additional records for this species and noted those specimens were “Deux exemplaires, dont un dans la collection François”. The four IRSNB specimens appear to have been identified by Fauvel and are labeled with Ile des Pins. However the last line of the label includes “f.Faustien”, which is a piece of information not noted in either of those publication by Fauvel. In a corrigenda and addendum by Fauvel (1903) he added “*Le frère Faustien a recueilli à l’île des Pins une collection importante, aujourd’hui réunie à la mienne par l’acquisition de la collection Hustache*.” Based on this, the specimens I had available for study would have been added to Fauvel’s collection long after he described *Homalosoma griseolum* in 1882. The whereabouts of the holotype is unknown.

#### Generic distribution.

 New Caledonian precinctive.

#### New Caladonian species.

*Sphodrosomus saisseti* Perroud and Montrouzier 1864 [type MNHN!]

*Sphodrosomus monteithi* Will 2006 [type MNHN!]

*Sphodrosomus griseolum* (Fauvel 1882) new combination [type not found]

=*Homalosoma griseolum* Fauvel 1882

#### Notes.

 The placement of *Setalidius griseolum* in *Sphodrosomus* is primarily supported by shared features of the male genitalia and female reproductive tract (Figs 38-39, 41-42). The genus *Homalosoma* is part of the *Trichosternus* series of Moore (1965) and those taxa have a distinct set of characteristics that have not been found in any New Caledonian carabids.

## Key to adults of Pterostichini and Loxandrini of New Caledonia

**Table:** 

1	Metepisternum much longer than wide (Fig. 43A), flight wings full	2
1'	Metepisternum short, scarcely longer than wide (Fig. 43B), flight wings reduced	5
2(1)	Proepisternum longitudinally strigose (Fig. 44A); abdominal ventrites withtransverse sulci (Fig. 44B)	*Darodilia* sp.
2'	Proepisternum smooth; abdominal ventrites without sulci	3
3(2')	Elytra intervals 2 or 3 with one or more dorsal punctures	*Pseudoceneus numeensis*
3'	Elytra intervals without dorsal punctures. (*Platycaelus*)	4
4(3')	Elytra and proepisterna smooth, dorsal color black with a more or less evident spectral iridescence or with only a subtle metallic reflex	*Platycaelus prolixus*
4'	Elytra and proepisterna finely punctulate throughout, color usually distinctly metallic dorsally	*Platycaelus melliei*
5(1')	Abdominal ventrites without transverse sulci	11
5'	Abdominal ventrites with transverse sulci (Fig. 44B). (*Prosopogmus*)	6
6(5')	Hind angles of pronotum without obvious jag at the level of the basal seta (Fig. 45A)	7
6'	Hind angles of pronotum with obvious jag at the level of the basal seta (Fig. 45B)	10
7(6')	Pronotum without border along base	8
7'	Pronotum with base bordered from near hind angle to level of inner basal impression	9
8(7)	Elytral striae distinctly crenulate, but not deeply punctate, surface with purple reflex	*Prosopogmus irideus*
8'	Elytral striae not crenulate, not or shallowly punctate, surface black, slightly iridescent	*Prosopogmus koghisensis*
9(7')	Pronotum with outer basal impression	*Prosopogmus fortis*
9'	Pronotum without outer basal impression	*Prosopogmus savesi*
10(6')	Angular base of stria one present, pronotum with outer basal impression well impressed or at least shallowly marked with a convex region laterally	*Prosopogmus lescheni*
10'	Angular base of stria one absent, pronotum without outer basal impression, not convex laterad impression	*Prosopogmus aoupiniensis*
11(5)	Elytral epipleura and most of the ventral surface with short scattered pubescent	*Paniestichus subsolianus*
11'	Elytral epipleura glabrous, ventral surface glabrous except for the typical arrangement of a few long setae on various segments	12
12(11')	Head contrastingly shinier than relatively dull pronotum and elytra (Figs 17, 31). Elytral interval 7 sharply carinate from humerus to level of plica	13
12'	Dorsal luster various. Elytral interval 7 not raised above the level of other intervals OR raised (sharply carinate or notably raised and rounded) only in the basal half of the elytra and apically not raised or only very slightly more pronounced than other intervals at that level. In some *Abacophrastus* that have a relatively raised interval 7, the dorsal surface luster is shiny throughout and the head is not contrastingly duller (Fig. 30)	16
13(12)	Elytral interval 5 neither carinate nor raised, prosternal process glabrous	*Platysmodes gambeyi*
13'	Elytral interval 5 sharply carinate, or noticeably raised in the apical third (Fig. 46), prosternal process with seta at apex (*Abacoleptus*	14
14(13')	Elytral interval 5 sharply carinate in apical third, body-form relatively narrow and elongate (Figs 17A, B), base of pronotum slightly wider than base of elytra	15
14'	Elytral interval 5 raised and rounded in apical third, body-form relatively broad (Fig. 17C), base of pronotum equal to width of elytra base	*Abacoleptus curtus*
15(14)	Elytral interval 6 ended well before apex of 7, intervals 5 and 7 conjoined or separate	*Abacoleptus carinatus*
15'	Elytral interval 6 ended slightly beyond apex of 7, intervals 5 and 7 separate	*Abacoleptus paradoxus*
16(12')	Setae present at or near the pronotal hind angles; elytra without punctures in interval 3	17
16'	Setae lacking at pronotal hind angle; elytra with one or more setigerous punctures in interval 3	*Cerabilia* spp (Loxandrini, ~24 sp. n. to be treated elsewhere)
17(16)	Prosternal process glabrous	19
17'	Prosternal process with seta at apex (*Abacomorphus*)	18
18(17')	Body-length 20mm or more, entirely black dorsally or rarely with a slight hint of a metallic reflex	*Abacomorphus asperulus*
18'	Body-length 15-20mm, dorsally black with vividly purple, blue or green metallic luster or at least with a notable metallic reflex	*Abacomorphus caledonicus*
19(17)	Tarsi dorsally glabrous	26
19'	Tarsi dorsally with fine, sparse setae (*Abacophrastus*)	20
20(19')	Base of elytra with complete raised margin	21
20'	Base of elytra raised margin broadly interrupted at stria 6	*Abacophrastus hobbit*
21(20)	Anterior face of mesotibia near ventral edge with two setate	22
21'	Anterior face of mesotibia near ventral edge with row of five to seven setate	*Abacophrastus chapes*
22(21)	Eye moderate sized, dorso-ventral width of eye 1.5-1.8x width of gena below eye	23
22'	Eyes very large, dorso-ventral width of eye 2.5x width of gena below eye	*Abacophrastus megalops*
23(22)	Elytral striae complete and well impressed throughout their length. Elytral humeri prominent, angulate with sharp outward turned denticle	24
23'	Elytral striae shallowly impressed in apical third, 6^th^ strae basally and 7^th ^stria medially very shallow. Elytral humeri prominent and angulate but denticle bluntly rounded	*Abacophrastus carnifex*
24(23)	Size large, length 13mm or more. Antennae long, antennomeres 5-10 elongate, length about 3x width	25
24'	Size small, length 10mm or less. Antennae short, antennomeres 5-10 stout, length about 2x width	*Abacophrastus millei*
25(24)	With convex 10^th^ interval at level of plica and 3-4 apical setigerous punctures in 9^th^ interval	*Abacophrastus bellorum*
25'	With convex 10^th^ interval at level of plica and 3-4 apical setigerous punctures in 9^th^ interval	*Abacophrastus reflexus*
26(19)	Frons smooth; elytral interval 7 rounded and slightly raised near humerus; body form moderately narrow (Figs 36, 37, 40)	27
26'	Frons rugose; elytral interval 7 sharply carinate near humerus; very broad body form (Fig. 34)	*Euryabax colossus*
27(26)	Head, pronotum and elytra similarly shiny; elytra slightly iridescent (*Setalidius*)	28
27'	Head and pronotum contrastingly shinier than dull elytra (*Sphodrosomus*)	29
28(27)	Elytral intervals 4-5 notably convex and arcuate onto the elytral base, clypeo-ocular sulci scarcely impressed	*Setalidius nigerimus*
28'	Elytral intervals 4-5 slightly convex and straight onto the elytral base, clypeo-ocular sulci elongate, impressed and divergent	*Setalidius attenuatus*
29(27')	Body-length 21mm or larger, antennae very long, antennomere 5 length five times its width	28
29'	Body-length under 14mm, antennae of average length, antennomere 5 length three times its width	*Sphodrosomus griseolum*
30(29)	Anterior face of profemur with one medial and one basal seta; elytral dorsal surface scarcely papillose, relatively smooth in dorso-medial third	*Sphodrosomus monteithi*
30'	Anterior face of profemur with more than two setae, in most individuals arranged as a longitudinal row of 4-10 setae and cluster of 2-4 basal setae; elytral dorsal surface clearly and uniformly papillose throughout	*Sphodrosomus saisset*

**Figure 44.**
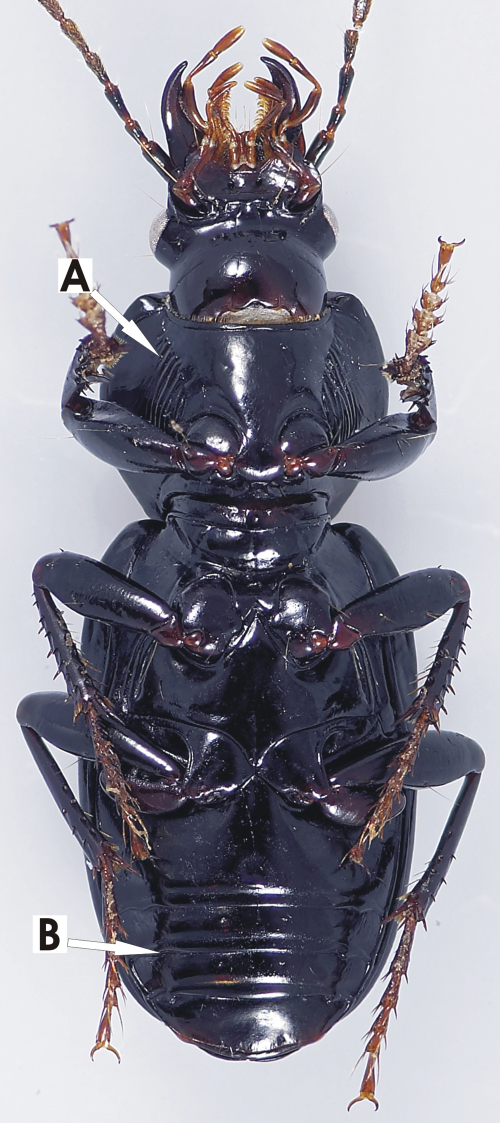
Ventral view, *Darodilia* undetermined species from New Caledonia showing **A** longitudinally strigose proepisternum **B** transverse sulci of abdominal sterna.

**Figure 45.**
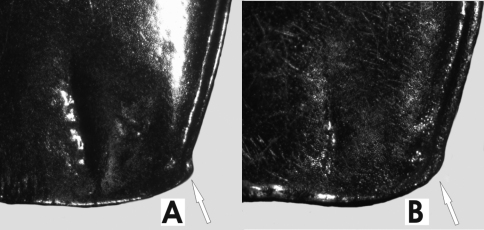
Right hind angle of pronotum showing **A** prominent jag in *Prosopogmus lescheni* **B** obtusely rounded angle in *Prosopogmus koghisensis*.

**Figure 46.**
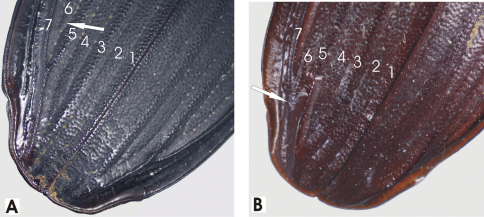
Apical portion of elytra showing **A** *Abacoleptus carinatus* with interval 6 ended well before apex of 7 **B** *Abacoleptus paradoxus* with interval 6 ended beyond apex of interval 7. Intervals are numbered and the arrows indicate the end of interval 6.

## Supplementary Material

XML Treatment for Platycaelus

XML Treatment for Darodilia

XML Treatment for Pseudoceneus

XML Treatment for Prosopogmus

XML Treatment for Prosopogmus koghisensis

XML Treatment for Prosopogmus lescheni

XML Treatment for Prosopogmus fortis

XML Treatment for Prosopogmus aoupiniensis

XML Treatment for Paniestichus

XML Treatment for Paniestichus subsolianus

XML Treatment for Abacoleptus

XML Treatment for Abacoleptus curtus

XML Treatment for Platysmodes

XML Treatment for Cerabilia

XML Treatment for Abacomorphus

XML Treatment for Abacophrastus

XML Treatment for Abacophrastus millei

XML Treatment for Abacophrastus chapes

XML Treatment for Abacophrastus carnifex

XML Treatment for Abacophrastus hobbit

XML Treatment for Abacophrastus megalops

XML Treatment for Abacophrastus bellorum

XML Treatment for Abacophrastus reflexus

XML Treatment for Euryabax

XML Treatment for Setalidius

XML Treatment for Sphodrosomus
